# A milestone in C4 carbon concentration mechanism evolution: structural remodeling of NADP-malic enzyme in Poaceae

**DOI:** 10.1093/molbev/msag056

**Published:** 2026-04-22

**Authors:** Jonas M Böhm, Simone Willms, Oja Ferrao, Martin Buitrago-Arango, Meike Hüdig, Gereon Poschmann, Nazanin Fazelnia, Luitgard Nagel-Steger, Sebastián Klinke, Athina Drakonaki, Christos Gatsogiannis, Marcos A Tronconi, Clarisa E Alvarez, Veronica G Maurino

**Affiliations:** Molecular Plant Physiology, Institute for Cellular and Molecular Botany (IZMB), University of Bonn, Kirschallee 1, Bonn 53115, Germany; Molecular Plant Physiology, Institute for Cellular and Molecular Botany (IZMB), University of Bonn, Kirschallee 1, Bonn 53115, Germany; Molecular Plant Physiology, Institute for Cellular and Molecular Botany (IZMB), University of Bonn, Kirschallee 1, Bonn 53115, Germany; Molecular Plant Physiology, Institute for Cellular and Molecular Botany (IZMB), University of Bonn, Kirschallee 1, Bonn 53115, Germany; Molecular Plant Physiology, Institute for Cellular and Molecular Botany (IZMB), University of Bonn, Kirschallee 1, Bonn 53115, Germany; Molecular Proteomics Laboratory, Biomedical Research Centre (BMFZ) and Institute of Molecular Medicine, Proteome Research, Medical Faculty and University Hospital Düsseldorf, Heinrich Heine University Düsseldorf, Universitätsstraße 1, Düsseldorf 40225, Germany; Institut für Physikalische Biologie, Heinrich Heine University Düsseldorf, Universitätsstraße 1, Düsseldorf 40225, Germany; Institut für Physikalische Biologie, Heinrich Heine University Düsseldorf, Universitätsstraße 1, Düsseldorf 40225, Germany; Fundación Instituto Leloir, IIBBA-CONICET and Plataforma Argentina de Biología Estructural y Metabolómica PLABEM, Av. Patricias Argentinas 435, Buenos Aires C1405BWE, Argentina; CryoEM of Complex Nanosystems, Center for Soft Nanoscience and Institute of Medical Physics and Biophysics, Busso-Peus-Str. 10, Münster 48149, Germany; CryoEM of Complex Nanosystems, Center for Soft Nanoscience and Institute of Medical Physics and Biophysics, Busso-Peus-Str. 10, Münster 48149, Germany; Centro de Estudios Fotosintéticos y Bioquímicos (CEFOBI-CONICET), University of Rosario, Suipacha 570, Rosario 2000, Argentina; Centro de Estudios Fotosintéticos y Bioquímicos (CEFOBI-CONICET), University of Rosario, Suipacha 570, Rosario 2000, Argentina; Molecular Plant Physiology, Institute for Cellular and Molecular Botany (IZMB), University of Bonn, Kirschallee 1, Bonn 53115, Germany

**Keywords:** C4 photosynthesis, cryo-electron microscopy, gene duplication, NADP-malic enzyme, protein oligomerization, structural evolution

## Abstract

The evolution of C4 photosynthesis required extensive modification of ancestral enzymes enabling the development of an efficient carbon concentrating mechanism. A key example is NADP-malic enzyme (NADP-ME), which, in maize and sorghum—members of the same C4 lineage—underwent gene duplication and neofunctionalization, resulting in 2 plastidic isoforms with distinct oligomeric states: a tetrameric C4-specific isoform and a dimeric housekeeping (nonC4) isoform. In this study, we resolve the structural basis of this oligomeric divergence using X-ray crystallography, cryo-electron microscopy, and molecular modeling combined with targeted biochemical analysis. Our findings demonstrate that the N-terminal region of nonC4-NADP-ME is involved in its oligomeric organization, whereas a suite of adaptive substitutions at the dimer interface drives the transition to the stable tetramer characteristic of the C4 isoform. Moreover, the C-terminal region stabilizes the oligomeric states of C4- and nonC4-NADP-ME through specific interactions with adaptive residues. We propose that tetramerization mitigates aggregation at the high expression levels demanded by the C4 cycle and likely creates a scaffold for the emergence of regulatory properties. Collectively, the data show that remodeling of terminal domains and inter-subunit interfaces rewires the quaternary architecture of the enzymes, illustrating how subtle structural changes can drive the evolution of complex innovations such as C4 photosynthesis.

## Introduction

The evolution of photosynthesis in land plants reveals a fascinating adaptation: the C4 carbon concentrating mechanism (Sage et al. 2012). This remarkable metabolic pathway enhances photosynthetic efficiency by concentrating CO_2_ around ribulose-1,5-bisphosphate carboxylase-oxygenase (Rubisco), which reduces its wasteful oxygen fixation reaction. The C4 pathway has independently evolved in at least 65 different lineages across 19 families of flowering plants (Sage et al. 2011). In most C4 lineages, NADP-malic enzyme (C4-NADP-ME), specifically localized in the chloroplasts of bundle sheath cells (BSC), catalyzes the decarboxylation of malate, thereby supplying a concentrated source of CO_2_ for Rubisco (Furbank 2011). This specialized C4-NADP-ME evolved from a plastidic nonphotosynthetic isoform (nonC4-NADP-ME) through gene duplication and subsequent modifications in the promoter and 5′ region, enabling high expression levels in BSCs, and further protein neo-functionalization (Tausta et al. 2002; Christin et al. 2009; Brown et al. 2011; Alvarez et al. 2019). The plant NADP-ME family is diverse, including housekeeping isoforms involved in essential processes such as defense responses (Maurino et al. 2001; Chi et al. 2004; Voll et al. 2012), seed germination (Detarsio et al. 2008; Yazdanpanah et al. 2019), and lipid biosynthesis (Lai et al. 2002) within both the cytosol and plastids (Maurino et al. 2009; Tronconi et al. 2018).

Amino acid substitutions that enabled C4-NADP-ME to participate in the C4 pathway are specific to genetically close C4 lineages (Alvarez et al. 2019; Alvarez and Maurino 2023; Eckardt et al. 2024). In C4 plants like maize (*Zea mays*) and sorghum (*Sorghum bicolor*), members of the same C4 lineage within the subfamily Panicoideae, the plastidic C4-NADP-ME and housekeeping nonC4-NADP-ME isoforms bear significant kinetic and structural differences. Compared to the plastidic nonC4 isoform, C4-NADP-ME exhibits higher affinity for the substrate malate, an increased catalytic rate, and malate inhibition at pH 7.0 (Maurino et al. 2001; Maier et al. 2011). Malate inhibition likely prevents excessive depletion of this metabolite during the night, when the stromal pH drops to approximately 7.0–7.5, thereby protecting the plant from nocturnal carbon starvation and ensuring a rapid reactivation of Rubisco at dawn (Zell et al. 2010; Bovdilova et al. 2019). The oligomeric state of C4-NADP-ME is also different from the nonC4 isoform in both species. C4-NADP-ME forms a stable tetramer of around 250 kDa within the physiological chloroplast pH range of 7.0 to 8.0 (Alvarez et al. 2019; Bovdilova et al. 2019), whereas nonC4-NADP-ME predominantly exists as a dimer of 130 kDa, as observed through native PAGE (Saigo et al. 2013; Alvarez et al. 2019). In most organisms, NADP-ME exists in a predominant oligomeric state, typically forming dimers or tetramers with strong interactions at the dimer interface and weaker interactions at the tetramer interface (Xu et al. 1999; Yang et al. 2002a, 2002b; Alvarez et al. 2019; Grell et al. 2022).

Different structural elements of the protomers have been identified as key determinants of the oligomerization state from different organisms. In human mitochondrial NADP-ME, the C-terminal loop interacts with the neighboring dimer, promoting tetramer formation (Grell et al. 2022). Additionally, in the other human mitochondrial isoform, NAD(P)-ME, a C-terminal extension of 7 residues stabilizes the tetramer by interacting with both protomers of the opposing dimer (Xu et al. 1999). Plant NADP-MEs possess a longer N-terminal region, although of variable length, than that of human enzymes, and recent studies have linked this region to changes in their quaternary structure. For instance, the deletion of the first 20 amino acid residues from the mature dimeric maize nonC4-NADP-ME induces tetramer formation (Saigo et al. 2013; Alvarez et al. 2019). Interestingly, although the transit peptide of maize and sorghum plastidic NADP-ME isoforms originated from a single evolutionary event (Tausta et al. 2002; Christin et al. 2009), the C4 isoform contains a shorter N-terminal region due to a 15-residues deletion (Alvarez et al. 2019). Despite these insights, the precise function of the N-terminal region in regulating oligomerization remains unclear, partly due to its unresolved structure in crystallized C4-NADP-MEs (Alvarez et al. 2019). Beyond the N-terminal differences, previous work has identified 20 residues as potentially critical to the evolution of maize and sorghum C4-NADP-ME, based on their differential conservation between the C4 and nonC4 isoforms (Alvarez et al. 2019). Four of these residues have previously been shown to contribute to specific C4 characteristics of maize C4-NADP-ME, and can therefore be considered adaptive substitutions (Alvarez et al. 2019).

In this study, we aimed at elucidating the structural determinants that differentiate the oligomerization of C4- and nonC4-NADP-ME isoforms in maize and sorghum. We specifically examine the N- and C-terminal regions and conserved amino acid residues that drive the structural divergence. Our results demonstrate that mutations at the dimer interface and modifications to the N-terminus selectively alter the oligomeric states of plastidic housekeeping and C4-NADP-ME. These findings provide valuable insights into the evolutionary mechanisms that underpin the distinct oligomerization patterns of these plastidic isoforms.

## Results

### Oligomeric organization of C4- and nonC4-NADP-ME in maize tissues

Our previous crystallographic studies indicated that C4-NADP-ME from maize and sorghum forms a tetramer composed of a dimer of dimers (Alvarez et al. 2019). This tetrameric configuration was further validated by analytical ultracentrifugation (AUC) (Figure S1a; (Bovdilova et al. 2019)). In contrast, the AUC of nonC4-NADP-ME revealed a dynamic dimer–tetramer equilibrium, with a tendency to aggregate (Figure S1a). The distribution profile from AUC indicated that the oligomeric state of nonC4-NADP-ME is concentration-dependent, with higher protein concentrations promoting the formation of higher-order oligomers (Figure S1a). Consistently, native PAGE showed that tetrameric C4-NADP-ME migrates between the 445 and 146 kDa markers, whereas nonC4-NADP-ME predominantly migrates as dimers just below the 146 kDa marker (Figure S1b).

To further examine the oligomeric state differences between C4- and nonC4-NADP-ME, we analyzed maize extracts from etiolated leaves and roots using an in-gel NADP-ME activity assay after native PAGE. Protein bands exhibiting activity were excised and analyzed by mass spectrometry. C4-NADP-ME and 2 cytosolic isoforms (Cyt1-NADP-ME [Cyt1]; A0A804NE51 and Cyt2-NADP-ME [Cyt2]; B6TVG1) showed similar mobility to recombinant C4-NADP-ME, indicating they form tetramers (Figure S1b). In contrast, plastidic nonC4-NADP-ME displayed migration similar to the recombinant dimeric protein in both tissues (Figure S1b). These findings demonstrate that C4- and nonC4-NADP-ME have distinct oligomeric states both as recombinant proteins and following extraction from maize tissues.

### The N-terminal of nonC4-NADP-ME is involved in its oligomeric organization

**Figure 1:**
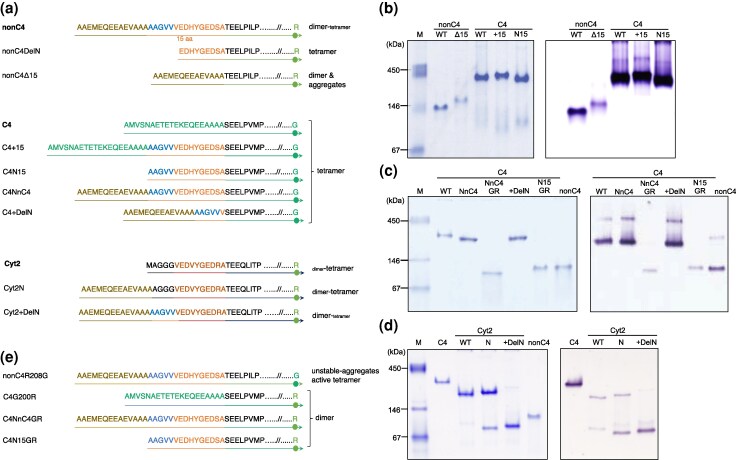
Analysis of C4- and nonC4-NADP-ME N-terminal variants. a) Overview of the chimeric and truncated N-terminal regions of the mature protein variants produced. b) Native PAGE (6%) of recombinant C4- and nonC4-NADP-ME variants with 5 μg protein loaded per lane, followed by a Coomassie staining (left), or with 3 μg protein loaded per lane, followed by an in-gel NADP-ME activity assay (right). c, d) Native PAGE (7%) of recombinant C4-NADP-ME (c) and Cyt2 (d) variants with 3 µg protein per lane, followed by a Coomassie staining (left), or with 1 μg protein loaded per lane, followed by an in-gel NADP-ME activity assay (right). In panels b–d, the SERVA native marker is shown on the left. e) Overview of the mutants produced at residue 200 in chimeric and truncated C4-NADP-ME variants and at the corresponding residue 208 in nonC4-NADP-ME.

Maize and sorghum nonC4-NADP-ME have a 15-residue (AAGVVVEDHYGEDSA) extended N-terminus that is absent in the C4 isoform (Fig. 1a). Previously, we have shown that by eliminating the first 20 N-terminal residues of maize nonC4-NADP-ME, which includes the first 6 residues (AAGVVV) of the 15-residue region (nonC4DelN; Fig. 1a), the oligomeric state shifted toward tetramers (Alvarez et al. 2019). Here, we further explore the role of the 15 residues in preventing tetramerization by generating several N-terminal deletion and insertion variants of the C4- and nonC4-isoforms (Fig. 1a). We found that all C4-NADP-ME variants (C4 + 15, C4N15, C4NnC4, and C4 + DelN) behaved similarly to the wild type (Fig. 1b, c), indicating the N-terminus does not affect its tetramerization. In contrast, removing the 15-residue N-terminus from nonC4-NADP-ME (nonC4Δ15) resulted in lower mobility in native PAGE (Fig. 1b) and protein aggregation (Figure S2b), highlighting the involvement of the N-terminal region in stabilizing nonC4-NADP-ME dimers.

To further investigate this, we expanded our analysis to a cytosolic NADP-ME from maize, whose ancestor is thought to have given rise to the plastidic isoforms by the acquisition of a transit peptide. Phylogenetic analysis indicated that the plastidic NADP-ME clade forms a sister group to a set of cytosolic isoforms (Figure S3), strongly suggesting that maize nonC4-NADP-MEs and Cyt2 share a common evolutionary origin. Comparative sequence analysis showed that Cyt2 has a shorter N-terminus than nonC4-NADP-ME, lacking the full N-terminal region of the nonC4 isoform until the end of the five-residue stretch AAGVV, resembling nonC4DelN (Fig. 1a). To investigate the structural and functional implications of the N-terminal differences between nonC4-NADP-ME and Cyt2, we expressed wild-type Cyt2 and generated 2 N-terminal modified variants (Fig. 1a). We found that wild-type Cyt2 predominantly forms tetramers, in a similar way as the nonC4DelN variant. In contrast, introducing the N-terminal region of nonC4-NADP-ME lacking the AAGVV sequence (Cyt2N) resulted in a mixture of tetramers and dimers, with tetramers being more prevalent (Fig. 1d). Furthermore, including the full N-terminal sequence with the AAGVV stretch (Cyt2 + DelN) induced a predominant dimer configuration, resembling the native oligomeric state of nonC4-NADP-ME (Fig. 1d). Collectively, these findings provide strong evidence that in nonC4-NADP-ME the N-terminal AAGVV sequence prevents tetrameric assembly.

### Residues mediating the quaternary-structure switch from nonC4- to C4-NADP-ME

Among the 20 adaptive substitutions that distinguish C4- from nonC4-NADP-ME, F140 is essential for quaternary-structure stability, whereas E339, Q503, and L544 enhance malate affinity without altering the enzyme conformation (Alvarez et al. 2019). To determine whether any of the remaining 16 substitutions also influence tetramer formation, we created a panel of C4-NADP-ME variants in which each of these positions was individually reverted to its nonC4 counterpart (Table S1).

**Figure 2:**
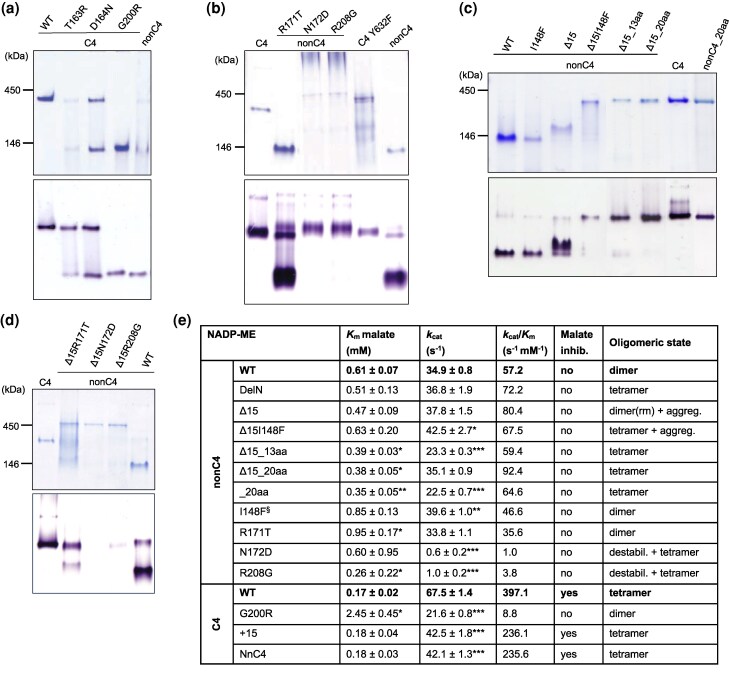
Native PAGE and kinetic analysis of C4- and nonC4-NADP-ME variants. a) Native PAGE (7%) analysis of recombinant C4-NADP-ME variants with single mutations in specific adaptive substitutions. Top panel: Coomassie staining with 3 μg protein per lane. Bottom panel: *in-gel* NADP-ME activity assay with 1 μg protein per lane. b) Native PAGE (7%) analysis of recombinant nonC4-NADP-ME variants with single mutations in specific adaptive substitutions. Top panel: Coomassie staining with 3 μg of C4- and nonC4-NADP-ME and 7.5 µg of mutant proteins per lane. Bottom panel: *in-gel* NADP-ME activity assay with 1 μg of C4 and nonC4-NADP-ME controls, and 2.5 µg of mutant proteins per lane. c) Native PAGE (7%) analysis of recombinant nonC4-NADP-ME variants with combined N-terminal modification and adaptive mutations. Top panel: Coomassie staining with 3 μg protein per lane. Bottom panel: *in-gel* NADP-ME activity assay with 1 μg of protein per lane. Due to low protein recovery, 2.5 μg of I148F and 1.5 μg Δ15 and Δ15I148F were loaded. In the nonC4-NADP-ME variant where 13 residues were simultaneously mutated (13aa), the enzyme contains the following mutations: F100T, I148F, H150N, D167N, R171T, N172D, E185V, R208G, Q209R, R274D, A347E, E511Q, and F552L. In the nonC4-NADP-ME variant where 20 residues were simultaneously mutated (20aa), the enzyme contains the following mutations: F100T, I148F, H150N, D167N, R171T, N172D, E185V, R208G, Q209R, R274D, H312D, I325F, A347E, V377M, H385Q, V482I, E511Q, N514T, D529A, and F552L (Table S1). d) Native PAGE (7%) analysis of recombinant nonC4-NADP-ME N-terminal variants, each with single mutations in specific adaptive substitutions as in B. Coomassie staining with 3 μg protein per lane. Bottom panel: *in-gel* NADP-ME activity assay with 1 μg protein per lane. e) Kinetic parameters of recombinant C4- and nonC4-NADP-ME wild type and mutant variants. Data were adjusted to the Michaelis–Menten equation by nonlinear regression with GraphPad Prism version 8.3.0 software (Boston, Massachusetts, USA). The values represent mean ± standard deviation; *n* = 3 independent enzyme preparations, each measured in triplicate. For statistical analysis, a two-tailed *t*-test with Welch's correction was performed, comparing nonC4- and C4-NADP-ME variants to the corresponding WT proteins. Asterisks indicate that the value is statistically significantly different from the corresponding WT. *P < 0.05; **P < 0.01; ***P < 0.001 (the *P* values are shown in Table S2). § values taken from Alvarez et al. (2019). rm denotes slightly reduced mobility with respect to the nonC4-NADP-ME.

Among 16 C4-NADP-ME mutants, 13 remained tetrameric and retained activity similar to the wild type after native gel electrophoresis (Figure S4). However, mutants C4T163R, C4D164N, and C4G200R exhibited altered oligomerization (Fig. 2a). Both C4T163R and C4D164N showed a dimer–tetramer equilibrium, with C4D164N comprising 48% dimers and 34% tetramers as confirmed by AUC (Figure S2). Notably, the C4G200R mutant formed a stable dimer, as evidenced by native PAGE (Fig. 2a) and AUC (Figure S2). We also generated the reverse mutants nonC4R171T, nonC4N172D, and nonC4R208G. The nonC4R171T mutant maintained a dimer-tetramer equilibrium highly displaced to the formation of dimers like the wild-type nonC4 isoform (Fig. 2b). By contrast, the nonC4N172D and nonC4R208G variants formed a small amount of enzymatically active tetramers, but they mainly aggregated into higher-order species, reducing soluble protein yield (Fig. 2b). These findings highlight the critical roles of residues 164 and 200 in C4-NADP-ME, and their counterparts in nonC4-NADP-ME, in regulating NADP-ME oligomer formation and stability.

Given the striking influence of G200 on the quaternary state of C4-NADP-ME, we further investigated its role in conjunction with N-terminal modifications. We produced 2 additional C4-NADP-ME variants: one with the N-terminal region of nonC4-NADP-ME extended by 15 residues and the G200R mutation (C4NnC4GR), and another with only the 15 residues at the N-terminus and the G200R mutation (C4N15GR) (Fig. 1e). In both cases, only active dimers were observed (Fig. 1c), indicating that the G200R mutation alone is sufficient to drive the shift in oligomerization from tetramers to dimers in C4-NADP-ME, and confirming that the N-terminal region of C4-NADP-ME is not directly involved in its tetramerization.

### Some adaptive residues override N-terminal control to promote tetramerization in nonC4-NADP-ME

Because oligomerization of nonC4-NADP-ME is influenced by both its N-terminal extension (Fig. 1b) and a suite of adaptive substitutions (Fig. 2b), we examined their combined effects. We engineered 2 protein variants lacking the first 15 residues (nonC4Δ15) that carried either 13 (Δ15_13aa) or all 20 (Δ15_20aa) adaptive substitutions. Both variants share 4 key mutations—I148F, R171T, N172D, and R208G (corresponding to F140, T163, D164, and G200 in C4-NADP-ME)—previously implicated in oligomer formation and stability. We found that both protein variants assembled into stable tetramers indistinguishable from C4-NADP-ME (Fig. 2c). Notably, introducing the full set of 20 adaptive substitutions into the intact nonC4-NADP-ME likewise drove tetramer assembly, indicating a dominant role of some adaptive mutations even in the presence of the native N-terminus (Fig. 2c).

To disentangle N-terminal effects from those of the 4 key residues or their interactions, we introduced single substitutions at I148, R171, N172, and R208 into the nonC4Δ15 scaffold, generating Δ15I148F, Δ15R171T, Δ15N172D, and Δ15R208G (Fig. 2c, d). In the Δ15 background, I148F and R171T shifted the equilibrium from dimers toward catalytically active tetramers, although some dimers and aggregation persisted (Fig. 2c, d; Figure S2). Introducing the same mutations into wild-type nonC4-NADP-ME left the oligomeric state unchanged (Fig. 2b, c), revealing epistasis between the N-terminus and these 2 residues for defining the oligomerization state. By contrast, combining Δ15 with either N172D or R208G drove the protein into high-molecular-weight aggregates, with only trace tetramer formation (Fig. 2d). When present alone in the wild-type nonC4-NADP-ME, each of these substitutions likewise promoted aggregation but still allowed a small tetramer population (Fig. 2b), indicating that in wild-type nonC4-NADP-ME N172 and R208 favor dimerization independently of the N-terminus. Nevertheless, the mutations alone are not enough to stabilize a tetrameric nonC4-NADP-ME. Importantly, in C4-NADP-ME the corresponding mutations D164N and G200R promote the formation of dimers independently from the N-terminal region (Fig. 2a), underscoring the crucial role of these residues in the evolutionary transition from nonC4- to C4-NADP-ME.

### The catalytic properties of NADP-ME isoforms are governed by epistatic interactions rather than the oligomeric state

To determine whether the oligomeric state of nonC4- and C4-NADP-ME correlates with the catalytic parameters, we conducted kinetic analyses on a series of enzyme variants carrying single and multiple amino acid substitutions.

In the nonC4Δ15 variant, which is dimeric, the deletion of 15 residues at its N-terminus does not alter the enzyme kinetic parameters with respect to the wild-type protein (Fig. 2e). Likewise, the tetrameric nonC4DelN variant retains wild-type kinetics (Fig. 2e; (Alvarez et al. 2019)). These findings indicate that the oligomeric state alone does not dictate the catalytic performance in nonC4-NADP-ME.

To further investigate this, we analyzed the nonC4Δ15I148F mutant, where the I148F modification introduced into the nonC4Δ15 background produces a tetrameric variant. This mutant shows increased catalytic rate without changes in malate affinity. However, additional mutations introduced in the nonC4Δ15I148F background (Δ15_13aa and Δ15_20aa) decreased catalytic rate to levels similar to or lower than nonC4-NADP-ME, while shifting malate affinity to values intermediate between those of the C4 and nonC4 enzymes (Fig. 2e). Notably, all these multiple mutants formed tetramers, reinforcing the notion that changes in oligomeric state do not directly explain the observed kinetic variations.

To dissect the contribution of individual residues, we next analyzed the kinetic properties of single nonC4-NADP-ME mutants targeting the 4 amino acids known to influence oligomeric organization. Among these, nonC4N172D and nonC4R208G, exhibited very low catalytic efficiency (Fig. 2e), likely due to limited formation of active tetramers (Fig. 2b). Interestingly, while nonC4N172D retained nonC4 wild-type malate affinity, nonC4R208G showed a malate affinity comparable to that of C4-NADP-ME. In contrast, the nonC4I148F and nonC4R171T dimeric variants displayed wild-type-like turnover rates (*k*_cat_) but much reduced malate affinity (Fig. 2e). Together, these results demonstrate that in nonC4-NADP-ME, kinetic properties are shaped by epistatic interactions among multiple amino acid residues. These interactions likely involve residues beyond the 20 differentially conserved ones, as evidenced by the nonC4Δ15_20aa and nonC4_20aa variants, which have a turnover rate similar or lower than wild-type nonC4-NADP-ME and have malate affinity intermediate between the nonC4 and C4 isoforms (Fig. 2e).

In C4-NADP-ME, insertion of the nonC4-specific N-terminal residues (in the C4 + 15 and C4NnC4 variants) does not affect malate affinity but reduces the turnover rate by 40% (Fig. 2e). This suggests that the shorter N-terminal region of C4-NADP-ME may contribute to its enhanced catalytic rate. Nevertheless, since both N-terminal mutants exhibit similar reductions in kinetic parameters yet fail to recapitulate the kinetic values of the nonC4-NADP-ME wild-type isoform, this points to epistatic interactions between the N-terminal region and other residues that are likely required to fully confer the isoform-specific properties.

Among the 4 residues implicated in oligomeric organization, only substitution at G200 produced a C4-NADP-ME variant with a dimeric, stable oligomeric state. The C4G200R mutant exhibited a 45-fold reduction in catalytic efficiency compared to the wild-type C4-NADP-ME, due to a 3-fold decrease in turnover rate and a 14-fold decrease in malate affinity. This pronounced effect could stem either from a structural role of G200 in tetramer stabilization or, despite its distance from the active site, a contribution to catalytic function. Additionally, the C4G200R mutant lost malate inhibition, in contrast to the C4 + 15 and C4NnC4 variants, which remain tetrameric and display wild-type-like malate sensitivity (Fig. 2e). These findings suggest a potential link between tetrameric structure and malate inhibition in C4-NADP-ME. However, this effect could also reflect a specific involvement of G200 in this regulation. Nevertheless, the reciprocal nonC4 mutant (nonC4R208G) and the nonC4_20aa variant do not gain malate inhibition. Further investigation is therefore required to clarify the mechanistic relationship between the G200 and malate inhibition in C4-NADP-ME.

Altogether, our results indicate that epistatic effects between residues are controlling the catalytic parameters independent of the oligomeric state in the NADP-ME isoforms.

### Residues involved in nonC4- to C4-NADP-ME oligomeric state shifts localize at the dimer interface

To pinpoint how isoform-specific residues influence oligomerization, we compared the dimer units of the 2 NADP-ME isoforms. One dimer came from the C4-NADP-ME crystal structure (PDB 5OU5), and 2 others were extracted from AlphaFold 3 tetramer models of the nonC4 and C4 isoforms (Figure S5). Superimposing the AlphaFold 3 dimers onto the crystal structure dimer yielded identical Cα RMSDs of 0.55 Å; just 0.08 Å arose from residues at the dimer interface, underscoring their near-perfect structural match. The remaining deviation was confined to catalytic domain C of protomer B, which alternates between “open” (nonC4) and “closed” (C4) conformations (Figure S5). This rigid-body movement of domain C is a conserved feature that gates catalysis in malic enzymes from diverse organisms (Xu et al. 1999; Chang and Tong 2003; Alvarez et al. 2019).

**Figure 3:**
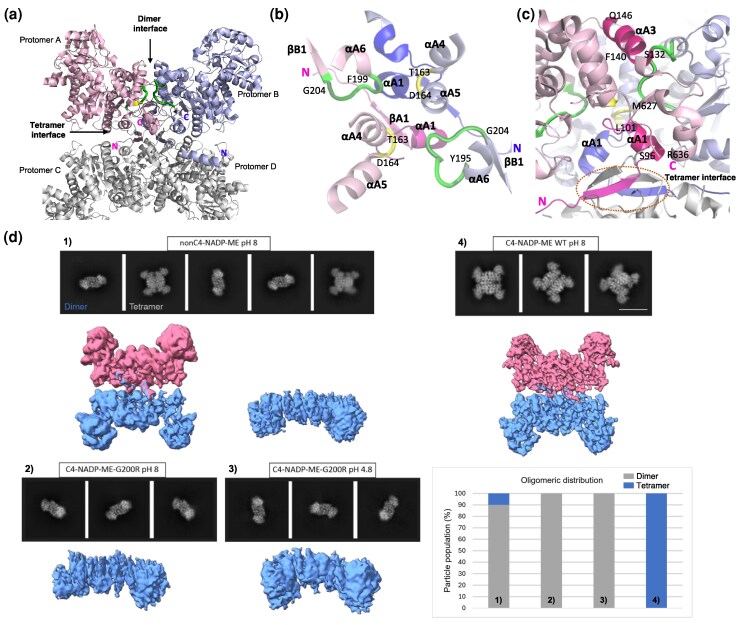
Structural analysis of C4- and nonC4-NADP-ME and C4G200R. a–c) Critical loops involved in the differential oligomeric states of C4- and nonC4-NADP-ME isoforms. a) Side view of the C4-NADP-ME structure (PDB ID: 5OU5), showing protomer A in pink and protomer B in purple. The C–D dimer is depicted in gray. Loops containing residues T163 and N164 are highlighted in yellow, while loops containing residue G200 are highlighted in green. The N- and C-termini of protomers A and B are shown. b) Top view of the dimer interface in C4-NADP-ME. Loops and formed by residues T163 and D164 are highlighted in yellow. Loops containing residues 199-FGRPQG-204 in protomer A and 195-YGSIFGRPQG-204 in protomer B are shown in green. N-termini are indicated. c) Detailed view of the C4-NADP-ME dimer interface, emphasizing the loops in proximity to helix αA3 and the N-terminal helix αA1. N- and C-termini of protomer A are indicated. The N-terminal β-sheet structures, which mediate interactions among all 4 protomers at the tetrameric interface, are enclosed within an oval. In b) and c) secondary structures of protomer A are shown in pink and those of protomer B in purple. d) Single particle cryoEM of nonC4-NADP-ME, C4G200R, and C4-NADP-ME. Representative 2D class averages and different views of cryo-EM volumes of nonC4-NADP-ME at pH 8.0 (a), C4G200R at pH 8.0 (b), C4-NADP-ME-G200R at pH 4.8 (c), and C4-NADP-ME at pH 8.0 (d), respectively (Scale bar: 110 Å). The distribution (%) of oligomeric state (dimer; gray, tetramer; blue) of each sample is shown based on the number of single particles in the reconstructions.

Furthermore, we found that the nonC4 and C4 isoforms feature 4 loops at the dimer interface (Fig. 3a, b). Two of these loops, one in each protomer, contain residues T163 and D164 in C4-NADP-ME (Fig. 3b) and the corresponding R171 and N172 in nonC4-NADP-ME (Figure S6), connecting helices αA4 and αA5 in each protomer (Fig. 3b; Video S1). The other 2 loops, also present in each protomer, vary in sequence and length between the isoforms, and feature G200 in C4-NADP-ME (Fig. 3b; Video S1; Figure S6) and the corresponding R208 in nonC4-NADP-ME (Figure S6). In both isoforms, the loop bridge helix αA6 with the βB1 sheet (Fig. 3b). Interestingly, the loops at the dimer interface of C4-NADP-ME feature 4 differentially conserved amino acid substitutions (T163, D164, G200, and R201; Figure S6) and are in close proximity to helix αA3 (Fig. 3c), which includes F140. In a previous work, we have shown that F140 does not interact directly with the neighboring protomer, but the adjacent residues K138 and K139 form hydrogen bonds with E288 and G196 of the other protomer, respectively (Alvarez et al. 2019). Additionally, near the loops at the dimer interface lies the C-terminal loop (M627-R636) and the N-terminal helix αA1 (S96-L101) of each of the 4 protomers (Fig. 3c; Video S1). Importantly, helix αA1 is positioned adjacent to the N-terminal β-sheet structures that mediate interactions between all 4 protomers at the tetramer interface in C4-NADP-ME (Fig. 3c; Video S1).

### Critical residues and interaction networks at the dimer interfaces of C4- and nonC4-NADP-ME isoforms

**Figure 4:**
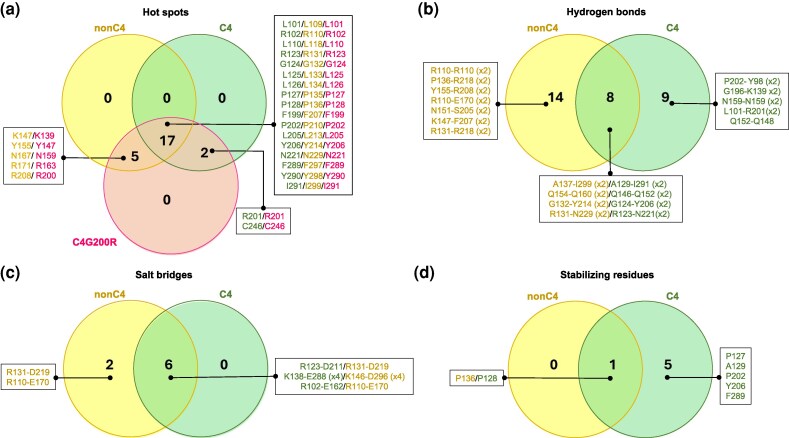
Hot spot and PISA A–B dimer interface comparison of nonC4- and C4-NADP-ME structures modeled by AlphaFold 3. a) Venn diagram summarizing the overlap of hot spot interface residues in the wild-type nonC4 and C4 isoforms and the C4G200R mutant. b) Residues that form hydrogen bonds across the protomers A and B dimer interface in nonC4- and C4-NADP-ME. c) Residues that participate in salt bridges between protomers A and B in nonC4- and C4-NADP-ME. d) Residues that contribute to dimer stabilization in nonC4- and C4-NADP-ME. ×2 and × 4 indicate that these residues are involved in interactions with 2 and 4 distinct atoms, respectively.

To identify critical interface residues essential for protomer interactions in C4- and nonC4-NADP-ME isoforms, we performed hot spot (HS) analysis employing the machine learning tool KFC2 (Zhu and Mitchell 2011). Comparative analysis of the dimeric structures modeled by AlphaFold 3 revealed 17 conserved HS residues (Fig. 4a). Additionally, residues K147, Y155, N167, R171, and R208 were uniquely identified as HS in nonC4-NADP-ME, while R201 and C246 were exclusive to C4-NADP-ME (Fig. 4a). Notably, the G200R mutation in C4-NADP-ME introduced 5 new HS (K139, Y147, N159, R163, and R200), corresponding to those uniquely present in nonC4-NADP-ME (Fig. 4a). Importantly, from these 5 residues, N167, R171, and R208 in nonC4-NADP-ME are among the 20 adaptive substitutions previously identified (Alvarez et al. 2019), with R171 and R208, along with their counterparts T163 and G200 in C4-NADP-ME, directly influencing the oligomeric state of the proteins (Fig. 2a, d; Video S1).

To gain a deeper understanding of the interactions at the dimer interface of nonC4- and C4-NADP-ME, we examined the interfaces of protomers A and B in both isoforms using PISA (Krissinel and Henrick 2007), focusing on hydrogen bonds (Fig. 4b and Table S4), salt bridges (Fig. 4c and Table S5), and interface stabilization (Fig. 4d; Table S6). C4-NADP-ME forms 17 H-bond interactions compared to 22 in nonC4-NADP-ME, with fewer than half conserved between the isoforms (Fig. 4b; Table S4). The isoform-specific hydrogen bond residues are primarily located in helices at the dimer interface and the interconnecting loops in both isoforms (Fig. 3b, c). Importantly, nonC4-NADP-ME uniquely forms hydrogen bonds between the nonC4 exclusive HS residues R208 and Y155 (Fig. 4b; Table S4).

Salt bridges analysis identified 6 interactions that are conserved in both isoforms. The nonC4-NADP-ME variant also harbors 2 additional specific salt bridges (Fig. 4c; Table S5). A single conserved proline provides further stabilization in each isoform (P136 in the nonC4 isoform, P128 in the C4 isoform; Fig. 4d; Table S6). The C4 isoform contains 5 extra stabilizing residues, among them P202 positioned at the “G200 loop” (Figure S6; Table S6; Video S1). Conversely, R201 within that same loop acts as a destabilizing residue (Table S6). In nonC4-NADP-ME, a destabilizing residue is R171 located in the “R171–N172 loop” (Table S6; Figure S6).

Overall, specific residues, particularly within the “G200 loop” in C4-NADP-ME and the “R171-N172 loop” in nonC4-NADP-ME, are pivotal in modulating dimer interface interactions.

### Crystallographic structure and oligomeric state analysis by cryo-EM of C4G200R

Given the crucial role of G200 in C4-NADP-ME oligomerization, we solved the C4G200R crystal structure at 2.7 Å resolution and pH 4.8 (see details of the crystal structure in Figure S7 and X-ray data collection in Table S7). The asymmetric unit contains a single homotetramer (dimer of dimers) with well-resolved electron density, except for the first 11 to 25 residues due to increased mobility. X-ray crystallography requires proteins to form a highly ordered crystal lattice, stabilizing a specific oligomeric state that may not be the predominant form in solution (Sala et al. 2020), which may explain the discrepancy with the native PAGE and AUC results.

To better capture the protein's oligomeric state in a more physiological setting, we conducted cryo-electron microscopy (cryo-EM), which visualizes proteins in a near-native, frozen-hydrated state (Maurino 2025). Consistent with our previous crystallographic studies, C4-NADP-ME exists exclusively as a tetramer comprising 2 dimeric subunits (Alvarez et al. 2019). In contrast, C4G200R forms dimers at both pH 4.8 and 8.0, whereas nonC4-NADP-ME primarily adopts a dimeric conformation, with a minor (10%) tetrameric population (Fig. 3d), corroborating the native PAGE results. These findings indicate that the C4G200R tetramer observed via X-ray crystallography at pH 4.8 is likely an artifact of crystallization rather than a physiologically relevant oligomeric state. The cryo-EM reconstructions suffer from anisotropic resolution due to the preferred orientation of particles and exhibit thus stretched densities; however, they allowed for rigid body fitting of the crystal structure (Figure S8). Oligomerization from dimer to tetramer does not involve large-scale conformational changes.

### Differential spatial positioning of residues at loop containing G200 in C4G200R promotes its dimerization

Comparison of the C4G200R mutant (PDB 9E6M) and the wild-type C4-NADP-ME (PDB 5OU5) crystal structures shows that the tetrameric framework is essentially unchanged (Figure S9a). The mutant buries ≈19,300 Å^2^ of surface at its subunit interfaces, slightly more than the ≈17,500 Å^2^ of the wild type. All 4 C4G200R protomers adopt the open conformation (Figure S9b), and their superposition yields Cα RMSDs values below 0.3 Å. In wild-type C4-NADP-ME crystal structure, protomers A, C, and D are likewise open, but protomer B is closed (Figure S9b); overlaying this closed protomer on all the open chains raises the Cα RMSD to 1.3 Å.

While the tetrameric assembly of C4G200R observed in the crystal is most likely due to lattice packing, we nevertheless probed whether the G200R substitution alters dimer–dimer interactions. Using PISA, we compared the A–C interface of the crystallographic C4G200R tetramer with that of the wild-type C4-NADP-ME. The G200R mutation causes a redistribution of hydrogen bonds, with the loss of 8 interactions present in the wild type and the formation of 4 new ones in the mutant (Figure S10a; Table S8). Despite these changes, the identity and positioning of stabilizing and destabilizing residues remain unaltered between both isoforms (Figure S10b, c; Table S9). Remarkably, C4G200R acquires 2 salt bridges between E217 and R636, which are absent in the wild-type C4-NADP-ME interface (Figure S10d; Table S10). In sum, the G200R mutation reconfigures part of the interaction network at the tetrameric interface.

**Figure 5:**
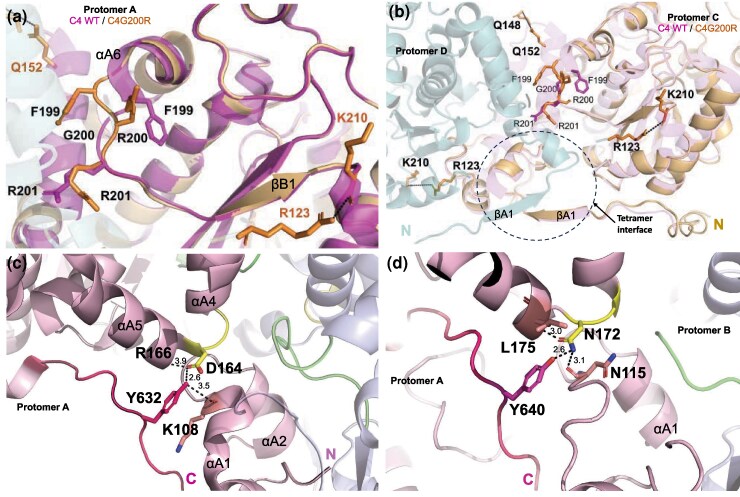
Loop conformations and tetramer interface interactions. a–b) Key structural differences between C4-NADP-ME and C4G200R. a) Comparative structural representation showing significant changes in the 199-FGRPQG-204 loop between maize C4-NADP-ME and C4G200R. Residues F199, R200, and R201 in C4G200R are depicted as orange sticks, while F199, G200, and R201 in C4-NADP-ME are represented as pink sticks. b) Relative positioning of the differential interactions identified by PISA analysis (Table S11) between C4-NADP-ME and the C4G200R. c, d) Interactions of the C-terminal end of C4- and nonC4-NADP-ME. c) Polar interactions of D164 with R166, Y632, and K108 at the tetrameric interface of C4-NADP-ME. d) Polar interactions of N172 with L175, Y640, and N115 at the tetrameric interface of nonC4-NADP-ME. In panels c and d, black dashed lines indicate polar bonds between the interacting functional groups, with bond distances denoted in Å.

Structural superposition of the C4G200R and wild-type C4-NADP-ME dimers pinpoints a local backbone shift confined to residues 199-200 (Fig. 5a, Video S2). The G200R substitution sits in the short “G200 loop” that bridges domain A (helix αA6) and domain B (strand βB1). To pinpoint the structural consequences of the G200R substitution, we used PISA to analyze the C–D dimer interfaces of C4-NADP-ME and its C4G200R variant, whose protomers C and D both adopt the open conformation. The analysis shows that the subtle re-orientation at the “G200 loop” reshapes the intersubunit contact map (Tables S11–13). The mutant gains 2 additional hydrogen bonds, 2 contacts between R123 and K210 (Video S2; Figure S11a; Table S11). By contrast, the hydrogen bond linking R201 and N159 in the wild-type enzyme is lost in C4G200R (Figure S11a; Table S11), indicating local rearrangements of the “G200 loop” that accompany the mutation.

At the C–D protomer dimer interface, 4 conserved stabilizing interactions are shared between C4-NADP-ME and C4G200R, whereas P127 is specific to C4-NADP-ME, and F129 and P202 are exclusive to C4G200R (Figure S11b; Table S12). Furthermore, R200, introduced in the G200R mutant, acts as a destabilizing residue, further differentiating the interaction profiles of the 2 isoforms (Figure S11c; Table S12). Three salt bridges at the C–D dimer interface are preserved between C4-NADP-ME and its C4G200R variant (Figure S11d; Table S13). The G200R mutation, however, abolishes 2 salt bridges: R102-E162, where R102 is a hotspot residue (Table S3) and 1 between K138-E288, and creates 2 new ones between R123 and D211 (Figure S11d; Table S13).

We then included nonC4-NADP-ME in the PISA analysis of the C–D dimer interfaces (Figure S12; Tables S14–S16). This analysis showed that the hydrogen bonds and the stabilizing or destabilizing residues gained in C4G200R (Figure S11a–c) are unique to the mutant and are not present in nonC4-NADP-ME (Figure S12a–c; Tables S14 and S15). Regarding salt bridges, 1 of the 3 bridges formed between D211 and R123 in C4G200R is also present in nonC4-NADP-ME (Figure S12d; Tables S16). In contrast, a salt bridge that is shared between C4- and nonC4-NADP-ME is absent in C4G200R, indicating that this interaction is lost in the mutant.

Altogether, this analysis shows that the incorporation of R200 in C4-NADP-ME introduces HS residues unique to nonC4-NADP-ME, highlighting the pivotal role of the G200-containing loop in mediating isoform-specific interactions and regulating the oligomerization state of the plastidic NADP-ME isoforms. Moreover, PISA analysis of the interfaces shows that C4G200R does not simply revert to a canonical nonC4-like interface but forms a distinct dimeric contact surface.

### C-terminal interactions stabilize oligomeric states of C4- and nonC4-NADP-ME

Structural analyses show that loops containing G200, T163, and D164 in C4-NADP-ME and their counterparts in nonC4-NADP-ME are positioned near the N- and C-termini at the tetramer interface (Fig. 3c; Video S1). Using PyMOL, we identified distinct hydrogen bond networks in these regions: in C4-NADP-ME, D164 forms bonds with R166 and Y632, which also bonds with K108, located in the loop connecting αA1 and αA2 at the N-terminus (Fig. 5c, d). In nonC4-NADP-ME, N172 (positionally homolog of D164 in C4-NADP-ME) interacts with L175, Y640, and N115 (Fig. 5d). Notably, our analysis showed that the D164N mutation in C4-NADP-ME altered the protein oligomeric state, whereas the reciprocal N172D mutation in nonC4-NADP-ME disrupted oligomeric stability (Fig. 2a, b). These different interactions in both isoforms facilitate the convergence of the N- and C-termini of each protomer, bringing them closer together and clustering them centrally (Fig. 5c).

To further investigate the functional role of the C-terminal end in protein stability, we generated the C4Y632F mutant by replacing tyrosine with phenylalanine, thereby removing the hydroxyl group essential for interactions with D164. We found that the Y632F mutation disrupted the stability of the tetrameric assembly, leading to the formation of a mixture of oligomeric states (Fig. 2b), similar to the F140I mutation (Alvarez et al. 2019). These results highlight the essential role of C-terminal interactions in maintaining the oligomeric structure and functionality of both C4- and nonC4-NADP-ME isoforms.

## Discussion

### Molecular determinants that drive the quaternary structure switch

Structural mapping pinpoints 4 adaptive substitutions that anchor the C4-NADP-ME tetramer: F140I, T163R, D164N, and G200R. Reverting any one of these residues to the nonC4 state destabilizes the tetramer and yields dimers or mixed oligomers, even when the N-terminus is unaltered. In the nonC4 enzyme replacing I148 or R171 with their C4 counterparts shifts the dimer toward a tetramer only after the 15-residue N-terminal extension is removed, revealing epistasis between these interface amino acids and the N-terminus. Two additional nonC4 residues, N172 and R208, are uniquely potent: the single changes N172D or R208G drive tetramerization regardless of the N-terminus, underscoring their central role in the evolutionary leap to the C4 quaternary state. Structural comparison between C4-NADP-ME and the C4G200R mutant revealed altered spatial positioning of residues within the “G200 loop” in C4G200R, introducing HS characteristic of the nonC4 interface. The G200 residue therefore operates as a structural toggle, dictating whether the loop embraces a neighboring dimer (tetramer) in the C4 wild-type enzyme or folds back to favor a stand-alone dimer in the C4G200R mutant.

Furthermore, interactions involving conserved residues D164 in C4-NADP-ME and N172 in nonC4-NADP-ME and the C-terminal residue Y632 in C4-NADP-ME and Y640 in nonC4-NADP-ME, are crucial for maintaining oligomeric stability. This mechanism is analogous to the stabilization seen in human mitochondrial NAD(P)-ME through C-terminal extensions, highlighting a conserved strategy for oligomeric stability across species (Xu et al. 1999).

Sequence insertions and deletions play a pivotal role in oligomerization (Jiang and Blouin 2007; Akiva et al. 2008; Hashimoto and Panchenko 2010). In maize and sorghum, the nonC4-NADP-ME isoform possesses a 15-residue longer N-terminal extension that is absent from the tetrameric C4 isoform. Removal of these residues alters its oligomerization, promoting aggregation. Truncation of the first 20 N-terminus residues of nonC4-isoform (nonC4DelN) induced its tetramerization. Adding this complete N-terminal extension to the maize cytosolic isoform Cyt2 induced its dimerization (Cyt2DelN), but not when the residues AAGVV were absent (Cyt2 + N) (Fig. 1d). This confirms that the residues AAGVV within the extended N-terminus of nonC4-NADP-ME disrupt dimer-dimer interactions. Similarly, modifications to the N-terminus of pigeon liver NADP-ME resulted in effects on oligomerization (Chou et al. 1997).

Our study also demonstrates that the N-terminal region does not influence the oligomerization of C4-NADP-ME, showing that interface mutations have made tetramerization N-terminus-independent. However, kinetic analyses indicate that the deletion of the 15 residues during the evolution of C4-NADP-ME is likely involved in enhancing its catalytic efficiency, as the enzyme variants with longer N-terminal regions have 40% less turnover rate. Since both N-terminal mutants exhibit similar reductions in kinetic parameters yet fail to recapitulate the values of the nonC4 wild-type isoform, this points to epistatic interactions between the N-terminal region and other residues that are likely required to fully confer the isoform's specific properties.

### Significance of C4-NADP-ME tetramerization for C4 metabolism

Our findings show that maize and sorghum C4-NADP-ME maintain a stable tetrameric state across physiologically relevant pH ranges, with no evidence of pH-dependent shifts in oligomerization. In contrast, the nonC4-NADP-ME predominates as a dimer. This raises the question of what advantages are conferred by tetramerization during the evolution of C4-NADP-ME. The evolution of C4 photosynthesis has led to a pronounced upregulation of genes encoding C4 pathway enzymes, including NADP-ME (Aubry et al. 2011; Maier et al. 2011). At high expression levels, proteins face increased risks of aggregation and misfolding in the cellular environment. In response to this, they typically evolve distinct sequence and structural features that enhance their stability (Tartaglia et al. 2007). We propose that in C4-optimized NADP-ME, adaptive amino acid substitutions promoted the evolution of a stable tetrameric assembly, likely to reduce the risk of protein aggregation under high cellular concentrations (Miller et al. 1987; Jones and Thornton 1995). This hypothesis is supported by our observation that the nonC4-isoform is prone to aggregation at high protein concentrations (Figure S1a). Furthermore, we hypothesize that tetramerization may have been necessary for the emergence of regulatory properties. The C4 NADP-ME tetramer is inhibited by malate at pH 7.0, whereas the dimeric C4G200R mutant completely loses this regulation. This contrast suggests that the quaternary switch might have created the structural context in which this regulatory feature could evolve. Dissecting the structural elements that couple tetramer formation to pH-dependent malate inhibition remains an open and promising avenue for future research.

Taken together, the stability advantage under high expression and the emergence of pH-dependent malate inhibition provide complementary selective pressures that can explain why tetramerization became fixed in maize and sorghum C4-NADP-ME.

### Evolutionary model for the evolution of distinct oligomerization states in plastidic NADP-ME isoforms

As the N-terminus is not involved in C4-NADP-ME tetramerization, which evolutionary scenario might have driven the loss of the 15 residues at the N-terminal of this isoform? A plausible hypothesis is that this truncation was associated with altering the expression pattern of the C4 isoform in specific tissues to meet the requirements of the C4 pathway. Unlike nonC4-NADP-ME, which is expressed across a diverse range of cell types and organs (Maurino et al. 2001; Alvarez et al. 2013), C4-NADP-ME is exclusively localized to BSC chloroplasts (Maurino et al. 1997; Swift et al. 2024). This tissue-specific expression is controlled by cis-regulatory elements in the 5′ coding regions that interact with trans-acting factors unique to C4 plants (Patel et al. 2004, 2006; Brown et al. 2011; Borba et al. 2023). These cis-elements, conserved across angiosperm monocots and eudicots, were repurposed during C4 evolution to drive BSC-specific expression, as seen in both maize and Cleome (Brown et al. 2011; Kajala et al. 2012; Swift et al. 2024). Interestingly, these cis-elements are also present in C3 plant orthologs, where the necessary trans-factors are absent (Brown et al. 2011; Kajala et al. 2012; Williams et al. 2012). Alignment of C4 and nonC4-NADP-ME from maize and sorghum, along with Cyt2 from maize and C3/C4-NAD-ME sequences, showed that nonC4-NADP-ME and Cyt2 retain all conserved cis-elements, inherited from a common cytosolic ancestor (Figure S13). However, in nonC4-NADP-ME, these elements are positioned further upstream of the ATG start codon because the 5′ region includes an additional 45 nucleotides that encode the 15-amino-acid extension at the isoform's N-terminus (Figure S13). Proper positioning of cis-elements is important for the BSC-specific *NAD(P)-ME* gene expression (Brown et al. 2011). As nonC4-NADP-ME transcripts are absent in BSCs despite the presence of cis-elements (Tausta et al. 2002), the increased distance between the cis-regulatory elements and the ATG start codon may therefore reduce or even suppress gene expression in these cells. However, it is also possible that additional factors, likely located in the promoter region, contribute to the broader expression pattern of nonC4-NADP-ME. In support of this, recent research demonstrated that sorghum DOF transcription factors activate BSC expression by binding to the sorghum C4-NADP-ME promoter, which is enriched in DOF motifs (Borba et al. 2023; Swift et al. 2024). This suggests that a combination of regulatory elements may play a role in modulating the expression of the plastidic NADP-ME isoforms in different cell types. Beyond potentially disrupting BSC-specific expression, we demonstrated that the first few nucleotides encoding the residues AAGVV in the extended N-terminal region of nonC4-NADP-ME enable this isoform to form a dimeric structure. This dimerization may have allowed the isoform to acquire new functional roles within the unique biochemical environment of C4 plants.

**Figure 6:**
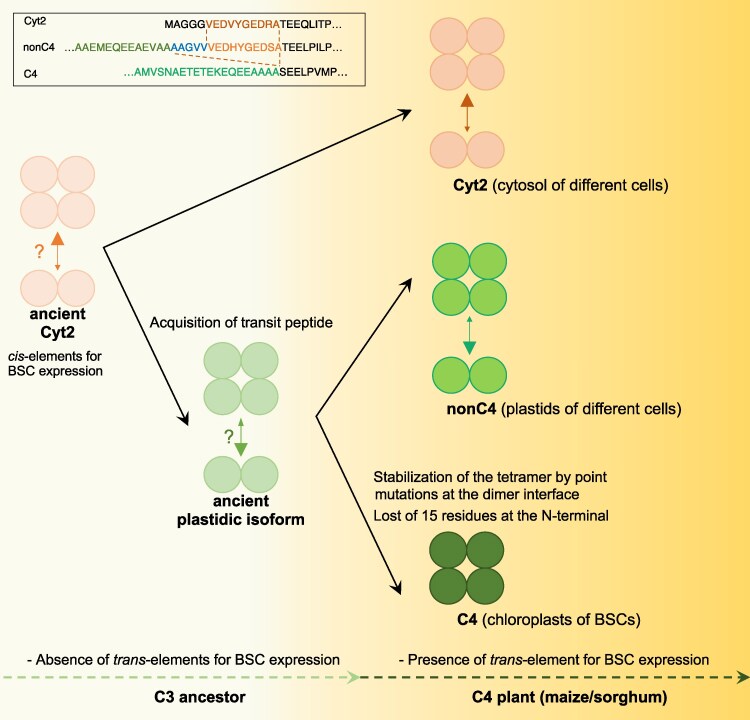
Proposed evolution of the oligomeric assembly of plastidic housekeeping (nonC4) and photosynthetic (C4) NADP-ME in maize and sorghum. The inset in the upper left corner shows the N-terminal sequences of maize Cyt2, nonC4-NADP-ME, and C4-NADP-ME isoforms. Arrow sizes correspond to the relative prevalence of each oligomeric state, with question marks indicating the most probable state of the ancient isoforms. The scheme was designed as a free-form artistic illustration, with the lines not representing branch lengths.

Our findings support a model for the evolution of distinct oligomerization states in plastidic NADP-ME isoforms within the same C4 lineage in Andropogoneae (Fig. 6). In the C3 ancestor, a gene duplication event involving an ancient cytosolic isoform (ancient Cyt2) gave rise to an ancient plastid-localized form, facilitated by the acquisition of an N-terminal transit peptide. A portion of the N-terminal region is conserved in the mature protein after peptide cleavage and contains amino acids critical for dimerization. The coding sequence of this N-terminus resides within a regulatory cluster that includes cis-elements necessary for specific expression of the protein in BSCs. With the evolution of C4 photosynthesis, a subsequent duplication of the gene encoding the ancestral plastidic isoform resulted in both the nonC4- and C4-NADP-ME isoforms, each retaining the transit peptide and cis-elements for BSCs expression. The C4-NADP-ME was adapted by evolving novel properties through specific amino acid substitutions and modifications at the N-terminal region. Likely, the 15-residue deletion in the N-terminal region of pre-C4-NADP-ME facilitated the proper arrangement of cis-elements, which is critical for the exclusive expression of C4-NADP-ME in BSCs.

Contrary to earlier hypotheses based on studies in sugarcane (Iglesias and Andreo 1990), our findings show that maize and sorghum C4-NADP-ME maintain a stable tetramer state across physiologically relevant pH ranges, without evidence of pH-dependent oligomeric transitions. This species-specific difference aligns with our previous analysis, which identified numerous C4-lineage-specific amino acid substitutions in C4-NADP-ME (Alvarez et al. 2019). Notably, the optimization of C4-NADP-ME has followed distinct evolutionary trajectories in different plant lineages, likely adapting to species-specific physiological contexts (Eckardt et al. 2024). These divergent evolutionary solutions to the same biochemical constraint have significant implications for metabolic engineering efforts aimed at introducing C4 photosynthesis into C3 plants (Ermakova et al. 2021). Successful engineering may require tailoring C4-NADP-ME variants to match the target cellular environment, as enzymes optimized for one species might not perform optimally in another.

## Methods

### Generation of expression constructs of C4-NADP-ME and nonC4-NADP-ME variants

To generate recombinant maize wild-type C4- and nonC4-NADP-ME, we used the expression vectors *pET16b::ZmC4-NADP-ME* and *pET28::ZmnonC4-NADP-ME*, which were constructed in our previous work (Alvarez et al. 2019). Expression vectors for various mutant variants of C4- and nonC4-NADP-ME were produced either through commercial gene synthesis by BioCat (Heidelberg, Germany), by site-directed mutagenesis (Table S17), or by restriction enzyme cloning (Table S17). In the case of commercial gene synthesis, the cDNAs were cloned into pET16b via NdeI/XhoI restriction sites.

### Site directed mutagenesis

Some mutant variants were generated by introducing point mutations into the expression vectors *pET16b::ZmC4-NADP-ME*, *pET28::ZmnonC4-NADP-ME*, or other commercially synthesized vectors (Table S17). To accomplish this, the entire expression construct was amplified by PCR using Phusion High-Fidelity DNA Polymerase (Thermo Fisher Scientific, Waltham, USA) and overlapping primers containing the desired nucleotide changes (see Table S18 for a list of primers used for site-directed mutagenesis). After PCR amplification, the template plasmid was removed by digestion with DpnI (Thermo Fisher Scientific, Waltham, USA) at 37 °C for 1 h. The digested PCR product was then purified using the Monarch PCR & DNA Cleanup Kit (New England Biolabs, Ipswich, USA) and used to transform *E. coli* DH5α. After plasmid purification, successful site-directed mutagenesis was confirmed by sequencing the expression vectors through EZ-seq services (Macrogen Europe, Amsterdam, The Netherlands).

### Restriction enzyme cloning

Classical restriction enzyme cloning was employed to produce *pET28::20aa_ZmnonC4-NADP-ME*. The vectors *pET28::ZmnonC4-NADP-ME* and *pET16b::*Δ15_20aa *ZmnonC4-NADP-ME* were digested consecutively with restriction enzymes (PasI/Xho1) and the resulting fragments were separated on an agarose gel. The vector fragments were extracted from the gel using the Monarch DNA Gel Extraction Kit (New England Biolabs, Ipswich, USA) and ligated with the T4 DNA Ligase (Thermo Fisher Scientific, Waltham, USA). The final vector was transformed into *E. coli* DH5α in order to amplify the plasmid, which was then confirmed via sequencing through EZ-seq services (Macrogen Europe, Amsterdam, The Netherlands).

### Generation of expression constructs of Cyt2 variants via Gibson assembly

To generate recombinant maize Cyt2 (B6TVG1), we used the *pET32a::ZmCyt2-NADP-ME* vector (Alvarez et al. 2013) as a template for PCR-based fragment construction, following the method described by Gibson (2011). For protein expression, Cyt2 and its N-terminal variants were cloned into the pET16b expression vector. For the Cyt2 construct, Gibson overhangs were incorporated in a single PCR reaction using primers complementary to both the fragment and the destination vector (Table S18). The resulting PCR product was purified using the Monarch PCR & DNA Cleanup Kit (New England Biolabs, Ipswich, USA). For the Cyt2N variant, the first 14 residues of nonC4-NADP-ME were fused to the N-terminal of Cyt2. This additional sequence was introduced in 2 consecutive PCR reactions with Phusion High-Fidelity DNA Polymerase (Thermo Fisher Scientific, Waltham, USA). After each PCR, the product was purified using the Monarch PCR & DNA Cleanup Kit. Primers were designed with one portion annealing to the PCR fragment and the other containing an overhang corresponding either to the additional nucleotide sequence or to the destination vector (Table S18). In the first PCR, part of the additional sequence was incorporated, while the second PCR introduced the remaining sequence along with the Gibson overhangs.

In the Cyt2DelN variant, the first 19 residues of nonC4-NADP-ME were fused to and partially replaced the N-terminal region of Cyt2. This nucleotide modification was introduced through 3 consecutive PCR reactions (Table S18) using Phusion High-Fidelity DNA Polymerase (Thermo Fisher Scientific, Waltham, USA). After each PCR step, the resulting fragments were purified using the Monarch PCR & DNA Cleanup Kit. In the first PCR, the original N-terminal sequence was mutated by introducing 2 nucleotide substitutions and adding 3 additional nucleotides. In the second PCR, as described for the Cyt2N variant, the first portion of the additional nucleotide sequence was added. The purified fragment was then inserted into a TOPO vector using the Zero Blunt TOPO PCR Cloning Kit (Thermo Fisher Scientific, Waltham, USA). After amplification in *E. coli* DH5α, the vector was sequenced to confirm the successful mutation of the N-terminus. The TOPO vector was then used as a template for the final PCR step, where the remaining nucleotide sequence and Gibson overhangs were added.

The empty pET16b vector was linearized with XhoI, and the purified fragments were inserted into the vector using Gibson isothermal assembly (Gibson 2011). The assembly reaction was carried out using reagents from Thermo Fisher Scientific (Waltham, USA) and New England Biolabs (Ipswich, USA). The resulting construct was transformed into *E. coli* DH5α, and correct insertion into the pET16b vector was verified by sequencing.

### Heterologous expression of NADP-ME variants

Expression constructs were transformed into chemically competent *E. coli* Rosetta2 (DE3; pLysSRARE2) cells (Merck, Darmstadt, Germany) or ArcticExpress (DE3) cells (Agilent Technologies, Santa Clara, USA). Transformed cells were plated on lysogeny broth (LB) agar plates containing 100 µg/ml ampicillin (for pET16b) or 50 µg/ml kanamycin (for pET28b) to select for the presence of the respective plasmids.

For the expression of His-tagged proteins, pre-cultures were grown overnight in LB medium containing the appropriate antibiotics at 37 °C, shaking at 140 rpm. The following day precultures were used to inoculate 400 ml of main cultures and further grown under the same conditions until an absorbance at *λ* = 600 nm (OD600) of 0.4 to 0.6 was reached, at which point protein expression was induced by adding 1 mM isopropyl-ß-D-thiogalactopyranoside (IPTG). Cells were harvested 2 h after induction. For expression of Cyt2 variants, cultures were grown at 37 °C with shaking at 140 rpm until an OD_600_ of 0.4 to 0.6 was achieved. The temperature was then lowered to 19 °C, and protein expression was induced by adding 1 mM IPTG. After 16 h of induction under these conditions, cells were harvested. For arctic express cells, cultures were grown at 30 °C until an OD600 of 0.4–0.6 was reached, then cooled to 11 °C, and protein expression was induced with 1 mM IPTG. Cells were cultured at 11 °C for 24 h before harvesting. To harvest the cells, cultures were centrifuged at 6,000 × *g* for 10 min at 4 °C, and the pellets were stored at −20 °C until used.

### Purification of recombinant NADP-ME variants

His-tagged proteins were purified using gravity-flow immobilized metal ion affinity chromatography on nickel-nitrilotriacetic acid (Ni-NTA) agarose. Frozen cells were thawed on ice and resuspended in 20 mM Tris–HCl (pH 8.0) containing 500 mM NaCl, 5 mM imidazole, 2 mM phenylmethanesulfonyl fluoride (PMSF), and a spatula-tip amount of lysozyme. The resuspended cells were sonicated for 8 min (30-s intervals with 30-s pauses) and then centrifuged at 15,000 × *g* for 15 min at 4 °C. The supernatant was loaded onto a pre-equilibrated Ni-NTA agarose column (Qiagen, Hilden, Germany) with a buffer containing 20 mM Tris–HCl (pH 8.0), 500 mM NaCl, and 5 mM imidazole. The column was subsequently washed with this buffer containing increasing concentrations of imidazole (5, 40, and 80 mM). Proteins were eluted with 2 ml of elution buffer containing 20 mM Tris–HCl (pH 8.0), 500 mM NaCl, and 500 mM imidazole. Finally, the eluted protein was concentrated and rebuffered with 20 mM Tris–HCl (pH 8.0) and 5 mM MgCl_2_ at 4 °C using Amicon Ultra 0.5 ml Centrifugal Filters (50 kDa molecular weight cutoff) according to the manufacturer's instructions (Merck, Darmstadt, Germany).

### Removal of His-tag

For His-tag removal, concentrated proteins were stored in the buffer recommended by the protease manufacturer. His-tags were cleaved using 2 different proteases, depending on the expression construct. For the pET16b vector, Factor Xa protease (New England Biolabs, Ipswich, USA) was used, while for the pET28b vector, Thrombin (Merck KGaA, Darmstadt, Germany) was employed. The recognition sites for both proteases are encoded in the respective expression vectors and are positioned between the His-tag and the NADP-ME sequence. The amount of protease used was in accordance with the manufacturer's instructions, and cleavage was carried out at 4 °C for 16 h. After cleavage, the His-tag and protease were removed by filtering the protein samples through Amicon Ultra 0.5 ml Centrifugal Filters (50 kDa molecular weight cutoff). The purified proteins were then rebuffered and stored in buffer containing 20 mM Tris–HCl (pH 8.0) and 5 mM MgCl_2_ at 4 °C, or in the same buffer with 20% glycerol at −80 °C for long-term storage.

### Analytical ultracentrifugation

Sedimentation velocity experiments were carried out using a Proteome Lab XL-A analytical ultracentrifuge (Beckman-Coulter, Brea, CA, USA). All recombinant versions of C4-NADP-ME and nonC4-NADP-ME were assayed in 20 mM Tris–HCl buffer (pH 7.0 or 8.0) with the addition of 5 mM MgCl_2_. Final protein concentration was adjusted to 1.1 mg ml^−1^. Samples (16 µM) and corresponding buffer solutions were loaded into aluminum double-sector centrepieces separately and built up in a Beckman An-60 Ti rotor. Experiments were performed at 20 °C and a rotor speed of 35,000 rpm. Protein samples were monitored by UV absorbance at *λ* = 280 nm in a continuous mode with a radial resolution of 0.003 cm. In time intervals of about 2 min, scans of the radial concentration profile were collected until the protein was fully sedimented. Data were analyzed using the *c*(*s*) model in the software package SEDFIT (Schuck and Rossmanith 2000). For data analysis, a resolution of 0.1 S with a confidence level (*F*-ratio) of 0.95 was chosen for the appropriate *s*-value range within 0 to 20.0 S. Density and viscosity of the solvent had been calculated with the software Sednterp (Laue et al. 1992) from tabulated values; *ρ* = 0.99823 g cm^−3^ and *η* = 0.01009 g cm^−1^s^−1^. The calculated partial specific volume of the individual proteins was approximately 0.738 cm^3^ g^−1^. Sedimentation coefficients are reported as *s*_20, w_ values, ie normalized to 20 °C and water as a solvent. Graphic output was generated by Gussi (Version 1.4.2) (Brautigam 2015).

### Protein crystallization and X-ray data collection

For crystallization studies of C4G200R, its His-tag was removed. The protein was crystallized using an Art Robbins Gryphon robot (Sunnyvale, CA, USA) on Jena Bioscience MRC 2-well sitting drop crystallization plates (Jena, Germany). Drops consisted of 0.2 µl of protein solution at 10 mg/ml in weak buffer (20 mM Tris–HCl pH 8.0, 5 mM MgCl_2_, 40 mM pyruvate, and 2 mM NADP) and 0.2 µl of commercial screen solution. Plates were incubated at 4 °C and were manually imaged on a Formulatrix Rock Imager 1000 apparatus (Bedford, MA, USA). C4G200R crystals appeared with a solution consisting of 2 M (NH_4_)_2_SO_4_, 0.1 M Na acetate, pH 4.6. Samples were directly harvested from the robotic plates, cryo-protected in their respective mother liquors, added with 35% (*w*/*v*) glycerol, and flash-cooled in liquid nitrogen for X-ray analysis.

### Data processing, structure determination, and refinement

Native X-ray diffraction data were collected at the P13 beamline, operated by EMBL Hamburg at the PETRA III storage ring (DESY, Hamburg, Germany (Cianci et al. 2017)) using a Dectris EIGER X 16M detector (Baden, Switzerland). Indexing, integration, and reduction were performed with XDS (https://xds.mr.mpg.de/) and Aimless-CCP4 (Agirre et al. 2023), leaving 5% of the reflections apart for cross-validation. The G200R structure (2.70 Å resolution) was solved by molecular replacement as implemented in CCP4 Cloud with MrBUMP (Keegan and Winn 2008), using the coordinates of the wild-type enzyme (PDB code 5OU5) as a starting model, followed by automatic model building with ModelCraft (Bond and Cowtan 2022). Subsequent steps of manual model building and restrained refinement were carried out with Coot (Emsley et al. 2010) and Refmac5 (Murshudov et al. 2011), respectively. In the last cycles, a total of 6 pyruvate molecules and 29 water molecules were added to the model. The structure was validated with MolProbity (Chen et al. 2010) and with the validation module of Coot. Detailed information on data collection, refinement, validation, and protein data bank (PDB) deposition is shown in Table S7.

### Cryo-EM sample preparation, data collection, and processing

For cryo-EM, purified C4-NADP-ME, C4G200R, and nonC4-NADP-ME with His-tag were prepared in 20 mM Tris–HCl, 5 mM MgCl_2_, 40 mM pyruvate, and 2 mM NADP (pH 8.0), and, purified C4G200R was additionally prepared in 10 mM Tris–HCl, 2.5 mM MgCl_2_, 20 mM pyruvate, 1 mM NADP, and 50 mM Na acetate (pH 4.8). To prepare the grids for cryo-EM, 4 μl of each sample solution (0.2 to 0.5 mg/ml) was applied to glow-discharged UltrAuFoil R1.2/1.3 grids (Quantifoil Micro Tools). The grids were blotted for 4.5 s and plunged frozen in liquid ethane after 0.5 s drain time in 100% relative humidity at 13 ^o^C (Vitrobot Mark IV, Thermo Fisher Scientific). Automated datasets of the clipped grids were acquired using the EPU software with a Krios G4 Cryo-TEM (Thermo Fischer Scientific) at 300 kV, equipped with an E-CFEG, a Selectris X Energy Filter with a slit width of 10 eV, and Falcon 4i Direct Electron Detector.

Each dataset (Table S19) was pre-processed using CryoSPARC Live (Punjani et al. 2017). The gain-corrected, motion-corrected, dose-weighted, and CTF-estimated micrographs were exported to CryoSPARC (v4.6.0, (Punjani et al. 2017)) for further processing. Particles were initially blob- picked and subjected to several rounds of 2D classification. Classes containing contaminants were removed. The remaining particles were used to create templates to enhance the accuracy and efficiency of particle picking. The newly template-picked particles were subjected to new rounds of 2D classification to clean the dataset, with “good” particles selected for ab initio reconstruction and subsequent homogeneous refinement. Figures S14–17 display the processing workflow for each dataset.

### Plant growth conditions and protein extract preparation

Maize seeds were soaked in water overnight, then wrapped in Whatman paper and secured inside the neck of a glass flask filled with water. The plants were grown at room temperature, either in the dark or under natural light conditions. After 7 days, leaves and roots were harvested, rapidly frozen in liquid nitrogen, and ground into a fine powder. Protein extracts were then prepared following the protocol described by Hüdig et al. (2022).

### Protein quantification and gel electrophoresis

Protein concentration was determined using a modified amido black 10B precipitation method (Schaffner and Weissmann 1973), with bovine serum albumin as the standard for the calibration curve, as described previously (Hüdig et al. 2022). For native polyacrylamide gel electrophoresis (PAGE), proteins were separated on a nondenaturing 7% (*w/v*) polyacrylamide gel at 100 V and 4 °C. Protein bands were visualized either by Coomassie Brilliant Blue staining or through an in-gel NADP-ME activity assay.

### In-gel NADP-ME activity assay and immunological detection

NADP-ME activity was detected after native PAGE using an in-gel activity assay. The gels were first incubated in 50 mM Tris–HCl (pH 8.0) for 15 min at room temperature, followed by incubation in the NADP-ME activity assay solution, which contained 50 mM Tris–HCl (pH 8.0), 10 mM L-malate, 10 mM MgCl_2_, 0.5 mM NADP, 0.05% (*w/v*) nitro blue tetrazolium, and 150 µM phenazine methosulfate. After a brief wash with distilled deionized water (ddH_2_O), the gels were imaged. For immunoblotting assays, the separated proteins were transferred to a PVDF membrane (Roti-PVDF, Carl Roth, Karlsruhe, Germany). After blocking with 5% milk powder in TBS, the membranes were incubated for at least 1 h with a 1:7,500 dilution of Anti-His-Tag antibody coupled to horseradish peroxidase (Anti-His-HRP, Miltenyi Biotec, Bergisch Gladbach, Germany). After washing several times with TBST, the membranes were incubated with a 1:2,500 dilution of Goat Anti-Rabbit IgG Antibody HRP-conjugate (Merck, Darmstadt, Germany). Proteins were visualized by chemiluminescence with Pierce ECL Western Blotting Substrate (Thermo Fisher Scientific, Waltham, USA) on a LAS-4000 Mini Luminescent Image Analyzer (GE Healthcare Life Sciences).

### Mass spectrometry

Protein extracts were separated on a 7% (*w/v*) native polyacrylamide gel electrophoresis (PAGE), and NADP-ME activity was visualized using an in-gel activity assay. The assay solution contained 50 mM Tris–HCl (pH 8.0), 100 mM L-malate, 10 mM MgCl_2_, 5 mM NADP, 0.05% (*w/v*) nitro blue tetrazolium, and 150 µM phenazine methosulfate. Gel slices exhibiting NADP-ME activity were excised and incubated for 20 min in a solution of 30% ethanol, 10% acetic acid, and 60% ddH_2_O. The gel slices were then prepared for mass spectrometric analysis, following the protocol described in Hüdig et al. (2022). Briefly, proteins were reduced with dithiothreitol (DTT), alkylated with iodoacetamide, and digested with trypsin. Tryptic peptides were extracted from the gel and purified using solid-phase extraction on an HLB µ-elution plate (Waters). Peptides were then separated using an Ultimate3000 rapid separation liquid chromatography system (Thermo Fisher Scientific, Waltham, USA) equipped with a C18 column, as previously described (Prescher et al. 2021). The peptides were injected into an online-coupled Fusion Lumos mass spectrometer (Thermo Fisher Scientific, Waltham, USA) with a FAIMS device via a nano electrospray source. The mass spectrometer was operated in data-dependent positive mode, with the FAIMS compensation voltage set to −50 V. Survey spectra were recorded in the Orbitrap analyzer (resolution 120,000, automatic gain control target 400,000, maximum ion time 60 ms, scan range 375 to 1,500 m/z, profile mode). Precursor ions with 2 to 7 charges were isolated via the built-in quadrupole (isolation window 1.6 m/z), fragmented by higher-energy collisional dissociation (normalized collision energy: 35), and fragment spectra were recorded in the linear ion trap (scan rate: rapid, automatic gain control target 10,000, maximum ion time 150 ms, scan range: auto, centroid mode). The cycle time was set to 1 s, with dynamic exclusion of 1 min.

Mass spectrometry raw files were processed using Proteome Discoverer 2.4.1.15 for protein identification, based on the 63,255 protein sequences of the *Zea mays* reference proteome (UP000007305, downloaded from UniProt on October 19, 2023), as well as potential contaminants from MaxQuant (version 1.6.17.0, Max Planck Institute for Biochemistry, Planegg, Germany). The SequestHT search engine was used for the database search, with mass tolerances set to 10 ppm for precursor ions and 0.6 Da for fragment ions. Cysteine carbamidomethylation was set as a fixed modification, and methionine oxidation, N-terminal acetylation, and methionine loss were considered as variable modifications. Proteins and peptides were accepted at a 1% false discovery rate (FDR), validated based on peptide spectrum matches, and only proteins identified with at least 2 unique peptides were reported. Protein grouping was disabled, and all individual protein matches were reported.

To identify cytosolic NADP-ME, protein sequences from previously identified ones (Alvarez et al. 2013) were compared to the reference proteome, and the highest matching proteins were used for identification.

### Kinetic parameters and statistical analyses

NADP-ME activity, specifically the oxidative decarboxylation of L-malate, was determined spectrophotometrically by monitoring the formation of NADPH at 340 nm and 25 °C using a Tecan Spark plate reader (Tecan Group, Männedorf, Switzerland). The Michaelis constant (*K*_m_) for malate was determined at pH 8.0 by varying malate concentrations around the expected *K*_m_ value, while keeping other components at saturating concentrations. The standard reaction mixture contained 50 mM Tris–HCl (pH 7.0 or 8.0), 10 mM MgCl_2_, 0.5 mM NADP, 200 ng of protein, and varying concentrations of L-malate ranging from 0.01 to 40 mM. For each assay, 192 µl of the reaction mixture was prepared in a Greiner 96-well flat transparent plate (Thermo Fisher Scientific, Waltham, USA). After recording the baseline measurement, 8 µl of increasing concentrations of L-malate were added to each well to achieve a final assay volume of 200 µl. NADPH formation was monitored at 340 nm and 25 °C for 25 min. Kinetic measurements were performed the day after protein purification, with proteins stored overnight at 4 °C. Activity measurements were repeated at least 3 times with independently expressed and purified protein samples, with each measurement consisting of triplicate assays for each concentration. Analysis of malate inhibition was performed at pH 7.0, with the remaining conditions staying the same. Kinetic parameters were calculated as described by Alvarez et al. (2019), using data from 3 independently purified protein batches and triplicate measurements for each. The extinction coefficient (*ε*) of 6.22 mM^−1^ cm^−1^ at 340 nm for NADPH was used in the calculations. Data were fitted to nonlinear regression in GraphPad Prism version 8.3.0 software (GraphPad Software, Boston, Massachusetts, USA) using free concentrations of all substrates.

Statistical analysis of kinetic measurements was performed with GraphPad Prism 8.3.0 software using a two-tailed *t*-test with Welch's correction (*α* < 0.05), comparing nonC4- and C4-NADP-ME variants to the corresponding wild-type proteins. *P*-values are shown in Table S2.

### Phylogenetic analysis

Multiple sequence alignment was performed as described in Tronconi et al. (2020). Maximum likelihood analysis of the complete dataset was carried out using MEGA X (v.10.0.5). The fit of each model to the data was evaluated using the Bayesian Information Criterion, with the model yielding the lowest BIC score being selected as the most appropriate for describing the substitution pattern. The initial ML tree was generated automatically using the NJ and BIONJ algorithms, and branch lengths were optimized to maximize the likelihood of the dataset under the chosen evolutionary model. Heuristic searches were then conducted starting from this initial tree, employing the nearest neighbor method, where alternative trees differ in one branching pattern. The reliability of internal branches was assessed through 2,000 bootstrap resamplings. Bootstrap values of 50% to 69% were considered weakly supported, 70% to 84% as moderately supported, and 85% to 100% as strongly supported (Hillis and Bull 1993). The tree was saved in Newick format (.nwk), containing all relevant clade support values and branch length information, and visualized using FigTree v1.4.4 software.

### Analysis of interactions and residues involved in oligomerization

Analyses of hydrogen bonds (Tables S4, S8, and S11) and salt bridges (Tables S5, S10, and S13) between protomers of a dimer and identification of residues with stabilizing or destabilizing roles in oligomer formation (Tables S6, S9 and S12) were performed using the PISA program (CCP4; (Krissinel and Henrick 2007)). Critical residues involved in stabilizing the oligomeric structure were identified through hot spot analysis using the KFC2 program (Zhu and Mitchell 2011) (Table S3). Hydrogen bond analysis and quality figures were generated with the licensed PyMOL Molecular Graphics System, Version 2.4.0 Schrödinger, LLC.

## Supplementary Material

msag056_Supplementary_Data

## Data Availability

The data supporting the findings of this manuscript are available from the corresponding author upon reasonable request. The C4G200R crystallographic structure was deposited in the Protein Data Bank (PDB) under the accession code PDB ID 9E6M. The mass spectrometry proteomics data have been deposited to the ProteomeXchange Consortium via the PRIDE (Perez-Riverol et al. 2022) partner repository with the dataset identifier PXD059557.
