# Supplementary material for: A milestone in C4 carbon concentration mechanism evolution: structural remodeling of NADP-malic enzyme in Poaceae
Source: Mol Biol Evol. 2026 Apr 22;43(4):msag056. doi: 10.1093/molbev/msag056 (PMC13100509; doi:10.1093/molbev/msag056)
Supplement: msag056_Supplementary_Data [file msag056_supplementary_data.zip › Supplementary material_251218.docx]

**Supplementary material**

Supplementary Figure 1. Analytical ultracentrifugation and native PAGE analysis. A. Continuous sedimentation coefficient distribution for maize C4- and nonC4-NADP-ME at pH 8.0 or 7.0. The distribution profile for nonC4-NADP-ME shows variability in oligomeric states (dimers and tetramers) depending on the protein concentration. The protein concentration analyzed are indicated in the graphics. Data were analyzed using the c(s) model in the software package SEDFIT. B. Native PAGE (7%) analysis of 35 µg of protein extract from etiolated leaf and root tissue coupled to an *in-gel* NADP-ME activity assay. The excised protein bands and the corresponding identified NADP-ME isoforms are shown on the right. Recombinant C4- and nonC4-NADP-ME were used as controls (0.5 µg) and SERVA native marker is shown on the left. Bands with NADP-ME activity were analyzed by MS and the identified NADP-ME isoforms are listed on the right.

**
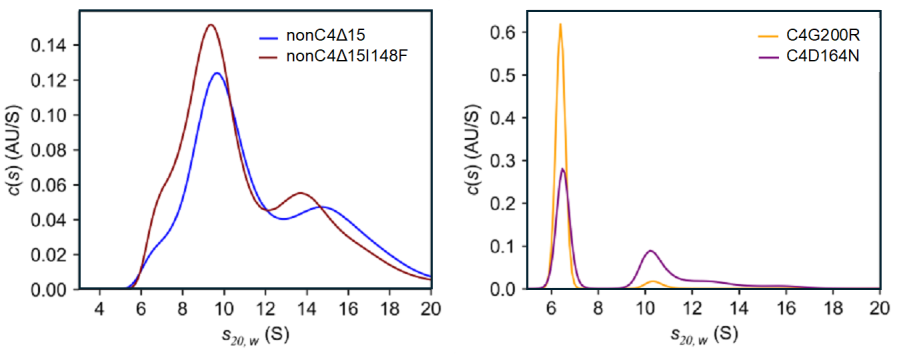
**

Supplementary Figure 2. Continuous sedimentation coefficient distribution of C4- and nonC4-NADP-ME variants. Measurements were conducted at pH 8.0. Data were analyzed using the c(s) model in the software package SEDFIT.

**Supplementary Figure 3. Phylogenetic tree of NADP-ME proteins in monocot.** The evolutionary relationships were inferred using the Maximum Likelihood (ML) method, based on a Multiple Sequence Alignment of 58 protein sequences spanning 554 amino acid positions from the genomes of 18 monocot species. *Acorus americanus*, an early-branching monocot, was used as the outgroup. The tree is drawn to scale, with branch lengths representing the number of substitutions per site. Evolutionary distances were calculated using the JTT matrix-based model, with a discrete Gamma distribution (parameter=0.52) to account for rate variation among sites. Bootstrap support values (from 2,000 replicates) are shown as MLB values next to branches, with values greater than 50% indicated. Cytosolic lineages I, II, and III are represented in black, gray, and blue, respectively. The plastidic lineage is shown in green. Maize and sorghum C4-NADP-ME are highlighted in dark green, while nonC4-NADP-ME are indicated in light green.

**Supplementary Figure 4. Native PAGE analysis of NADP-ME variants.** Native PAGE (7%) analysis of recombinant C4-NADP-ME variants. Top panel: Coomassie staining with 3 μg protein per lane. Bottom panel: *in-gel* NADP-ME activity assay with 1 μg protein per lane.


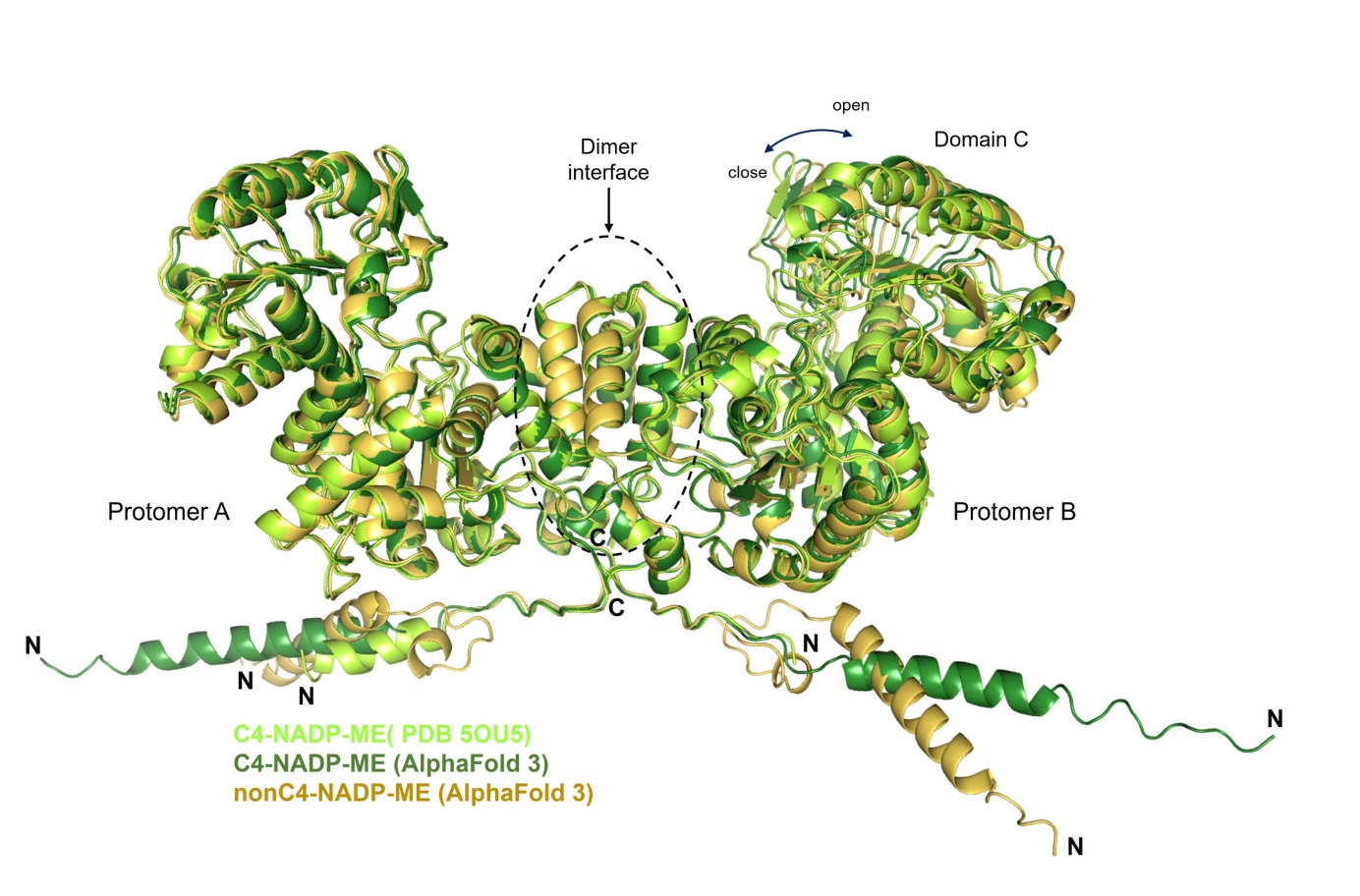


**Supplementary Figure 5. Structural comparison of nonC4- and C4-NADP-ME dimers.** Superposition of three dimeric models: AlphaFold 3 nonC4-NADP-ME (yellow), AlphaFold 3 C4-NADP-ME (light green) and the C4-NADP-ME crystal structure (dark green, PDB 5OU5). The structures share a highly conserved dimer interface (0.08 Å Cα RMSD), while the principal deviation, responsible for the bulk of the overall 0.55 Å Cα RMSD, arises from a rigid-body shift of catalytic domain C in protomer B, reflecting its open/closed conformational switch (arrow). The N-terminus (N) and C-terminus (C) of each protomer are indicated. Distinct N-termini are shown for each structure due to their different starting points and spatial predictions, while a single C-terminus is indicated, reflecting the convergence of all C-termini at the same site.

Supplementary Figure 6. Structural comparison of dimer interface loops in C4- and nonC4-NADP-ME. The yellow loops include residues T163 and D164 in C4-NADP-ME, corresponding to R171 and N172 in nonC4-NADP-ME. In C4-NADP-ME, the green loops feature residue G200. Specifically, in protomer A, the loop spans residues 199-204 (FGRPQG), and in protomer B, residues 195-204 (YGSIFGRPQG). In nonC4-NADP-ME, the corresponding green loops contain residue R208 and extend from residues 207-212 (FRQPQG) in protomer A to residues 206-213 (IFRQPQGL) in protomer B.

Supplementary Figure 7. Crystallography of C4G200R. A. Cartoon illustration of the crystallographic structure of C4G200R. Protomer A (pink) and protomer B (purple) form one dimer, while protomer C (green) and protomer D (yellow) form the second dimer. Pyruvate molecules are shown at each of the four active sites as space-filling spheres. The N-termini (N) and C-termini (C) above the 2D plane are labelled. For a detailed examination of all termini, please refer to the supplementary videos. B. Top-down view of C4G200R highlighting the tilted arrangement of the dimers relative to one another.

**Supplementary Figure 8. Rigid body fitting of the crystal structure into the cryoEM volumes.** The figure illustrates the rigid body fitting of the crystal structure into the cryo-EM maps for the nonC4-NADP-ME (pH 8), C4-NADP-ME-G200R (pH 8 and 4), and C4-NADP-ME (pH 8) variants.

**Supplementary Figure 9. Crystal-structure comparison of C4G200R and C4-NADP-ME.** **A.** Tetrameric superposition obtained by independently aligning each C4G200R dimer with its counterpart in C4-NADP-ME, thus compensating for the isoform-specific dimer-dimer tilt. Protomers are displayed as cartoons and individually coloured. Pyruvate molecules are shown at each of the four active sites as space-filling spheres. The N-termini (N) and C-termini (C) above the 2D plane are labelled. For a detailed examination of all termini, please refer to the supplementary videos. **B.** Overlay of the four C4G200R protomers shows that every subunit adopts the open conformation, coinciding with protomer A of C4-NADP-ME (light green) and contrasting with the closed protomer B of C4-NADP-ME (dark green). Blue arrows trace the displacement of domain C during the closed-to-open transition.

**Supplementary Figure 10.  PISA analysis of the A-C tetrameric interface of C4G200R and C4-NADP-ME crystal structures A.** Hydrogen bond network. **B.** Residues predicted to stabilize the interface. **C.** Residues predicted to destabilize the interface. **D.** Salt bridge contacts. Residues belonging to C4-NADP-ME are shown in green; those from C4G200R are shown in magenta. x2 and x3 denotes two independent atom-to-atom contacts for the same residue pair, respectively. A complete list of interacting atoms is provided in Supplementary Tables 8-10. All protomers were superposed in the open conformation of domain C so that the effect of the G200R substitution on the C-D interface could be assessed directly.

**Supplementary Figure 11. PISA analysis of the C-D dimer interface in C4-NADP-ME and C4G200R.** **A.** Hydrogen bond network. **B.** Residues predicted to stabilize the interface. **C.** Residues predicted to destabilize the interface. **D.** Salt bridge contacts. Residues belonging to C4-NADP-ME are shown in green; those from C4G200R are shown in magenta. x2 denotes two independent atom-to-atom contacts for the same residue pair. A complete list of interacting atoms is provided in Supplementary Tables 11-13. All protomers were superposed in the open conformation of domain C so that the effect of the G200R substitution on the C-D interface could be assessed directly.

**Supplementary Figure 12. PISA analysis of the C-D dimer interface of crystal structures of C4-NADP-ME (5OU5) and C4G200R (9E6M) in comparison with the AlphaFold 3 nonC4-NADP-ME model.** **A.** Hydrogen bond network. **B.** Residues predicted to stabilize the interface. **C.** Residues predicted to destabilize the interface. **D.** Salt bridge contacts. Residues belonging to C4-NADP-ME are shown in green; those from C4G200R are shown in magenta and nonC4-NADP-ME in yellow. x2 denotes two independent atom-to-atom contacts for the same residue pair. A complete list of interacting atoms is provided in Supplementary Tables 11-16. All protomers were superposed in the open conformation of domain C so that the effect of the G200R substitution on the C-D interface could be assessed directly.

**Supplementary Figure 13. Alignment of 5´coding sequences showing regions of identical nucleotide sequences shared between monocot and dicot NAD(P)-ME enzymes.** Predicted transit peptide cleavage sites are shown in bold. The sequence encoding the additional residues found in the N-terminal region of maize and sorghum nonC4-NADP-ME are shown in orange. Elements (a to s) and Box 1 and Box 2 are annotated according to Brown et al. (2011).


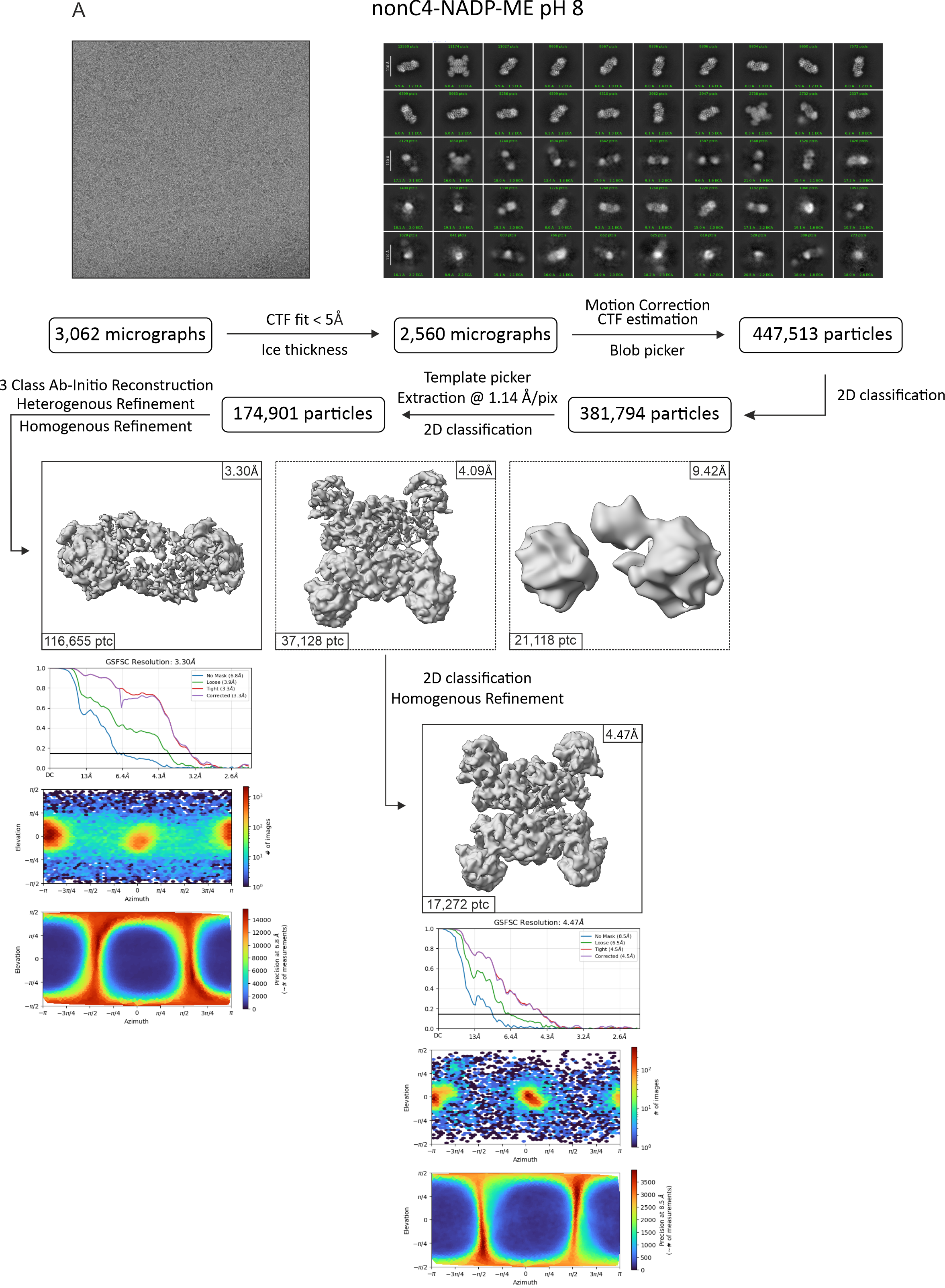


**Supplementary Figure 14. Cryo-EM analysis workflow of nonC4-NADP-ME, pH 8.** A representative micrograph, 2D class averages and flowchart for cryo-EM data processing are shown for nonC4-NADP-ME, together with half-map FSC curves and angular distributions for both oligomerization states. Note that the sample shows strong preferred orientation, represented by the angular distribution and the final reconstructions appear streaked perpendicular to the viewing direction (see also Figure 3).


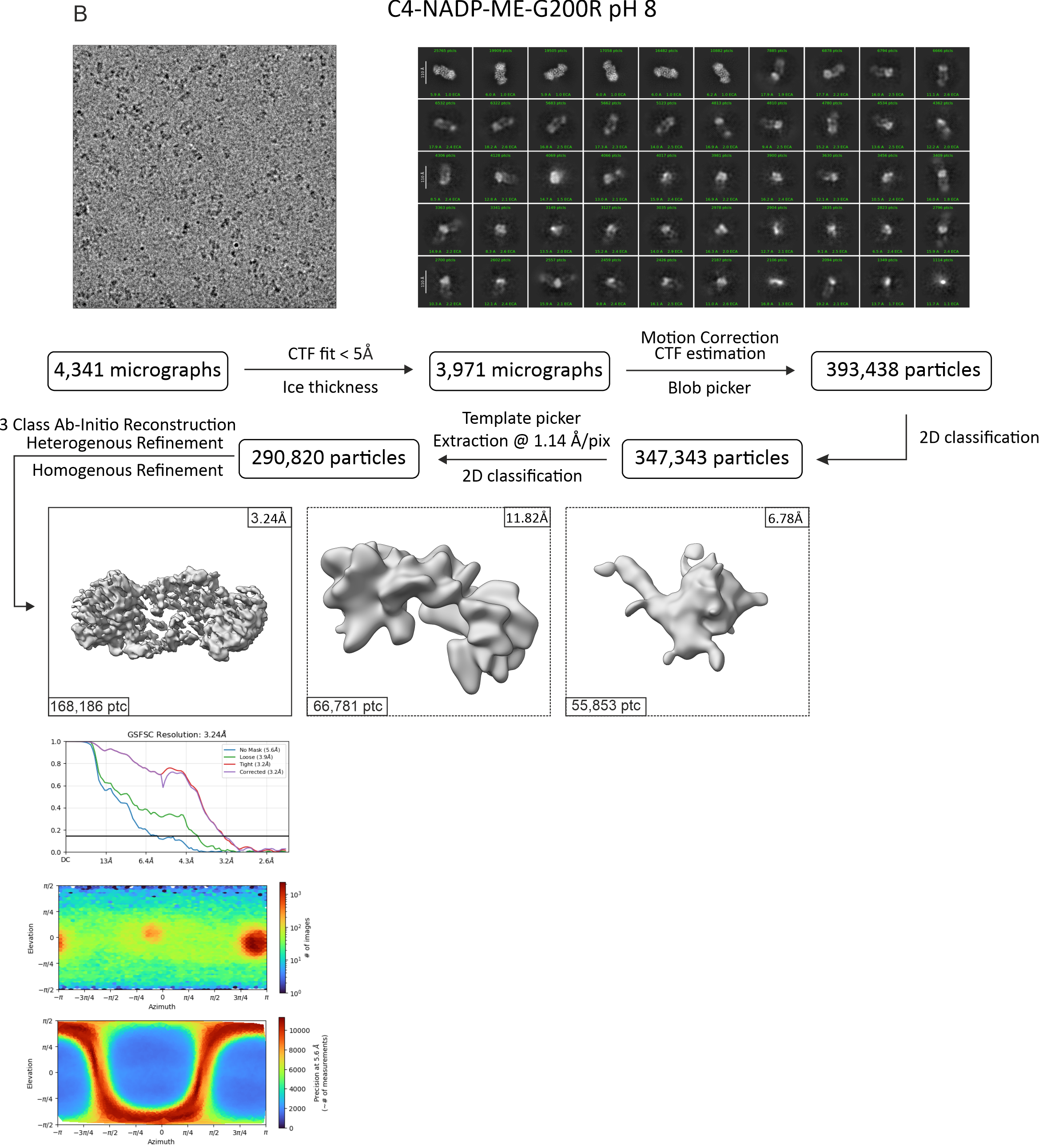


**Supplementary Figure 15. Cryo-EM analysis workflow of C4-NADP-ME-G200R, pH 8.** A representative micrograph, 2D class averages and flowchart for cryo-EM data processing are shown for C4-NADP-ME-G200R, pH 8, together with half-map FSC curves and the angular distribution. Note that the sample shows strong preferred orientation, represented by the angular distribution and the final reconstruction appear streaked perpendicular to the viewing direction (see also Figure 3).


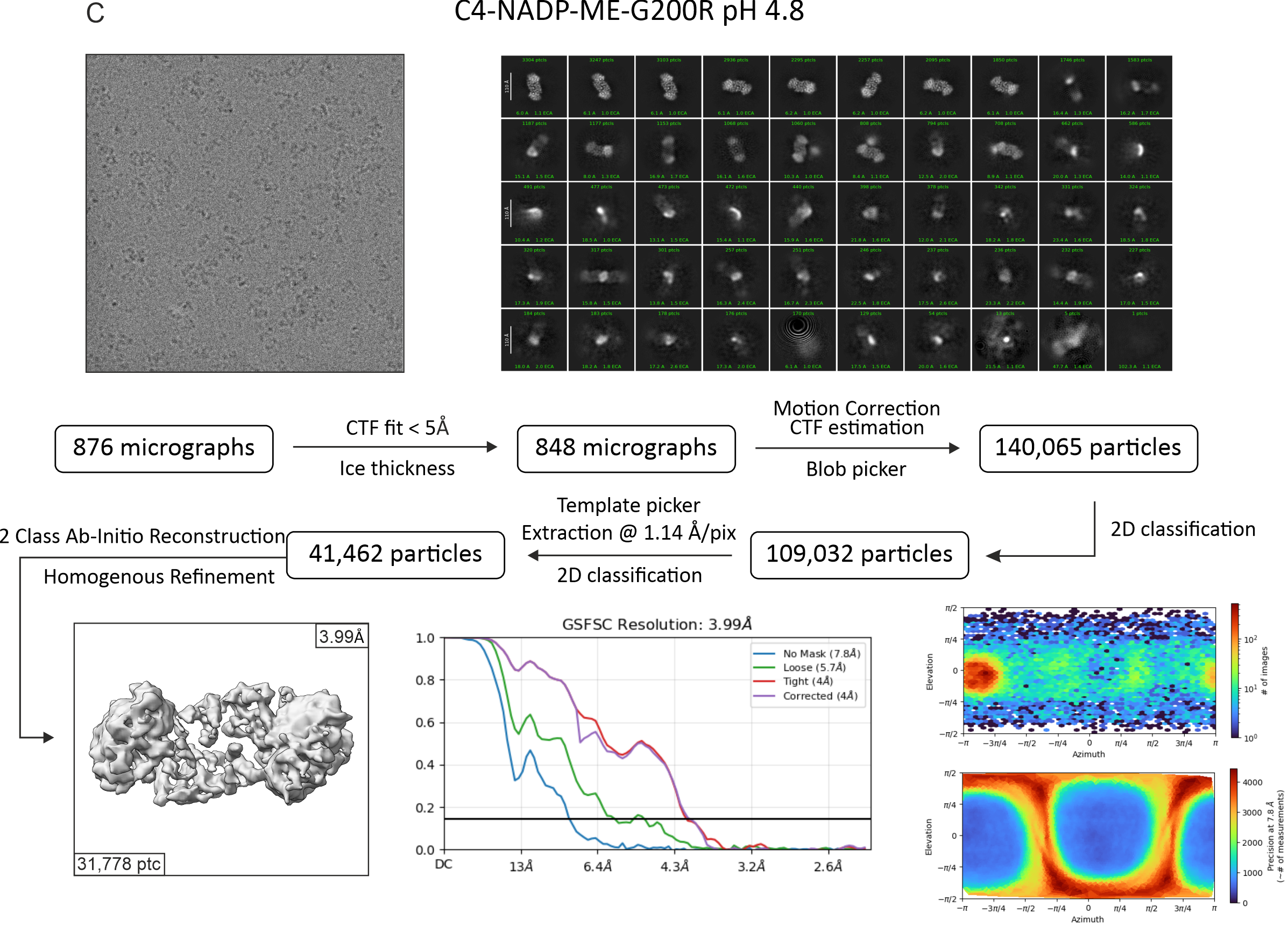


**Supplementary Figure 16. Cryo-EM analysis workflow of C4-NADP-G200R, pH 4.** A representative micrograph, 2D class averages and flowchart for cryo-EM data processing are shown for C4-NADP-ME-G200R, pH 4, together with half-map FSC curves and the angular distribution. Note that the sample shows strong preferred orientation, represented by the angular distribution and the final reconstruction appear streaked perpendicular to the viewing direction (see also Figure 3).


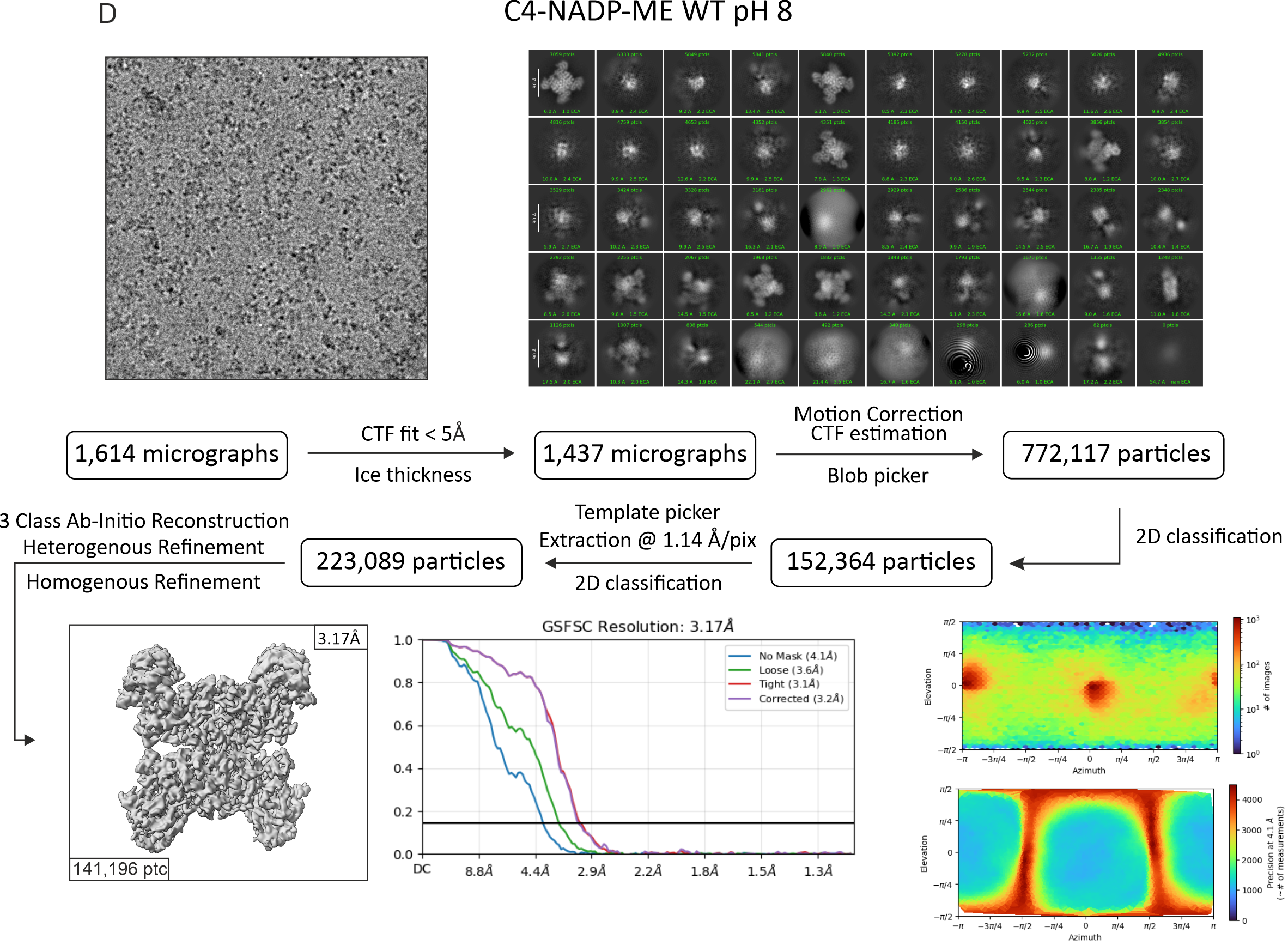


**Supplementary Figure 17. Cryo-EM analysis workflow of C4-NADP-ME, pH 8.** A representative micrograph, 2D class averages and flowchart for cryo-EM data processing are shown for C4-NADP-ME, pH 8, together with half-map FSC curves and the angular distribution. Note that the sample shows strong preferred orientation, represented by the angular distribution and the final reconstruction appear streaked perpendicular to the viewing direction (see also Fig. 3).

**Supplementary Table 1.** **Residues strictly differentially substituted between C4- and nonC4-NADP-ME in maize and sorghum as described in Alvarez et al. (2019).** Amino acid numbering corresponds to positions in the full-length sequence of maize C4-NADP-ME, and the positional homologous residues of the full-length nonC4-NADP-ME.

| **C4-NADP-ME** | **nonC4-NADP-ME** |
| --- | --- |
| T92 | F100 |
| F140 | I148 |
| N142 | H150 |
| N159 | D167 |
| T163 | R171 |
| D164 | N172 |
| V177 | E185 |
| G200 | R208 |
| R201 | Q209 |
| D266 | R274 |
| D304 | H312 |
| F317 | I325 |
| E339 | A347 |
| M369 | V377 |
| Q377 | H385 |
| I474 | V482 |
| Q503 | E511 |
| T506 | N514 |
| A521 | D529 |
| L544 | F552 |

**Supplementary Table 2. P-values from the kinetic parameters shown in Table 1.** P-values from two-tailed t-tests with Welch’s correction, comparing the kinetic parameters of nonC4-NADP-ME variants to the nonC4 wild-type enzyme and C4-NADP-ME variants to the C4 wild-type isoform. (*) P < 0.05; (**) P < 0.01; (***) P < 0.001. (ns) not statistically significant.

| **NADP-ME** | | **P-value *K*_m_** | **P-value *k*_cat_** |
| --- | --- | --- | --- |
| **nonC4** | DelN | 0.2927 (ns) | 0.2180 (ns) |
|  | Δ15 | 0.0933 (ns) | 0.0549 (ns) |
|  | Δ15I148F | 0.9056 (ns) | 0.0326 (*) |
|  | Δ15_13aa | 0.0171(*) | 0.0003 (***) |
|  | Δ15_20aa | 0.0101 (*) | 0.7492 (ns) |
|  | _20aa | 0.0064(**) | <0.0001 (***) |
|  | R171T | 0.0108 (*) | 0.1283 (ns) |
|  | N172D | 0.9704 (ns) | 0.0001 (***) |
|  | R208G | 0.0246 (*) | 0.0001 (***) |
| **C4** | G200R | 0.0126 (*) | <0.0001 (***) |
|  | +15 | 0.6552 (ns) | <0.0001 (***) |
|  | NnC4 | 0.7574 (ns) | <0.0001 (***) |

**Supplementary Table 3. Residues identified as hot spots (HS) using the KFC2 machine learning program.** HS exclusive for each protein are highlighted in bold. An asterisk, indicates the HS introduced by the G200R mutation in C4-NADP-ME (C4G200R).

|  | **nonC4-NADP-ME** | **C4-NADP-ME** | **C4G200R** |
| --- | --- | --- | --- |
| Leu | 109 | 101 | 101 |
| Arg | 110 | 102 | 102 |
| Leu | 118 | 110 | 110 |
| Arg | 131 | 123 | 123 |
| Gly | 132 | 124 | 124 |
| Leu | 133 | 125 | 125 |
| Leu | 134 | 126 | 126 |
| Pro | 135 | 127 | 127 |
| Pro | 136 | 128 | 128 |
| Lys | **147** | - | **139*** |
| Tyr | **155** | - | **147*** |
| Asn | **167** | - | **159*** |
| Arg | **171** | - | **163*** |
| Phe | 207 | 199 | 199 |
| Arg | **208** | - | **Arg 200*** |
| Gln | - | **Arg 201** | **Arg 201** |
| Pro | 210 | 202 | 202 |
| Leu | 213 | 205 | 205 |
| Tyr | 214 | 206 | 206 |
| Asn | 229 | 221 | 221 |
| Ser | - | **Cys 246** | **246** |
| Phe | 297 | 289 | 289 |
| Tyr | 298 | 290 | 290 |
| Ile | 299 | 291 | 291 |

**Supplementary Table 4. Hydrogen bond interactions between protomers A and B in C4- and nonC4-NADP-ME.** The table details the residues and atoms participating in the interactions detected by PISA, along with the corresponding distances (Å). Isoform-specific differences are color-coded: green for C4-NADP-ME and yellow for nonC4-NADP-ME. Residues labeled with ʆ indicate changes in amino acid identity between the isoforms, while residues marked with * belong to the loop containing G200 in C4-NADP-ME. Equivalent residues of each isoform are indicated.

| **Residue (Protomer A)** | **Atom** | **Distance (Å)** | **Residue (Protomer B)** | **Atom** | **Equivalent residue to:** | |
| --- | --- | --- | --- | --- | --- | --- |
| **C4-NADP-ME** | | | | | **nonC4-NADP-ME** | |
| P202(*) | O | 3.6 | Y98 | O^η^ | P210(*) | Y106 |
| I291 | O | 3.1 | A129 | N | I299 | A137 |
| G196(*) | O | 2.8 | K139 | N^ζ^ | G204 | K147 |
| Q146 | O | 3.2 | Q152 | N^ε2^ | Q154 | Q160 |
| N159($ʆ$) | O^δ1^ | 3.5 | N159($ʆ$) | N^δ1^ | D167($ʆ$) | D167($ʆ$) |
| N159($ʆ$) | N^δ2^ | 3.4 | N159($ʆ$) | O^δ1^ | D167($ʆ$) | D167($ʆ$) |
| L101 | O | 3.4 | R201(*)($ʆ$) | N^η1^ | L109 | Q209(*) ($ʆ$) |
| L101 | O | 3.7 | R201(*)($ʆ$) | N^η2^ | L109 | Q209(*) ($ʆ$) |
| G124 | O | 2.8 | Y206 | N | G132 | Y214 |
| R123 | O | 3.0 | N221 | N^δ2^ | R131 | N229 |
| N221 | N^δ1^ | 2.9 | R123 | O | N229 | R131 |
| Y206 | N | 2.8 | G124 | O | Y214 | G132 |
| Q152 | N^ε2^ | 3.2 | Q146 | O | Q160 | Q154 |
| Q152 | N^ε2^ | 3.5 | Q148 | O^ε1^ | Q160 | Q156 |
| K139 | N^ζ^ | 2.8 | G196(*) | O | K147 | G204 |
| Y98 | O^η^ | 3.4 | P202(*) | O | Y106 | P210(*) |
| A129 | N | 3.1 | I291 | O | A137 | I299 |
| **nonC4-NADP-ME** | | | | | **C4-NADP-ME** | |
| R110 | N^η1^ | 3.1 | R110 | O | R102 | R102 |
| N229 | N^δ2^ | 2.8 | R131 | O | N221 | R123 |
| Y214 | N | 2.6 | G132 | O | Y206 | G124 |
| R218($ʆ$) | N^η1^ | 3.8 | P136 | O | K210($ʆ$) | P128 |
| Q160 | N^ε2^ | 3.7 | Q154 | O | Q152 | Q146 |
| R208(*)($ʆ$) | N^η2^ | 3.7 | Y155 | O^η^ | G200(*)($ʆ$) | Y147 |
| R110 | N^η2^ | 3.2 | E170 | O | R102 | E162 |
| N151($ʆ$) | N^δ2^ | 2.8 | S205 | O | T143($ʆ$) | S197 |
| K147 | N^ζ^ | 2.9 | F207(*) | O | K139 | F199 |
| R131 | N^η1^ | 3.9 | R218($ʆ$) | O | R123 | K210($ʆ$) |
| R131 | O | 2.9 | N229 | N^δ2^ | R123 | N221 |
| A137 | N | 3.1 | I299 | O | A129 | I291 |
| R110 | O | 3.2 | R110 | N^η1^ | R102 | R102 |
| G132 | O | 2.6 | Y214 | N | G124 | Y206 |
| P136 | O | 3.8 | R218($ʆ$) | N^η1^ | P128 | K210($ʆ$) |
| Q154 | O | 3.7 | Q160 | N^ε2^ | Q146 | Q152 |
| Y155 | O^η^ | 3.7 | R208(*)($ʆ$) | N^η2^ | Y147 | G200((*)($ʆ$) |
| E170 | O | 3.3 | R110 | N^η2^ | E162 | R102 |
| S205 | O | 2.8 | N151($ʆ$) | N^δ2^ | S197 | T143($ʆ$) |
| F207(*) | O | 2.9 | K147 | N^ζ^ | F199 | K139 |
| R218($ʆ$) | O | 3.8 | R131 | N^η1^ | K210($ʆ$) | R123 |
| I299 | O | 3.1 | A137 | N | I291 | A129 |

**Supplementary Table 5. Salt bridge interactions between protomers A and B in C4- and nonC4-NADP-ME.** The table summarises every salt bridge contact detected by PISA, listing the interacting atoms and the corresponding distances (Å) for each residue involved. Atoms involved exclusively in one isoform are colour-coded - green for C4-NADP-ME and yellow for nonC4-NADP-ME - to emphasise isoform-specific contacts. Residues labeled with (¥) are located at the C-terminus of the αA1 helix, and those marked with (£) are situated within the αA3 helices in both isoforms. Equivalent residues of each isoform are indicated.

| **Residue (Protomer A)** | **Atom** | **Distance (Å)** | **Residue (Protomer B)** | **Atom** | **Equivalent residue to** | |
| --- | --- | --- | --- | --- | --- | --- |
| **C4-NADP-ME** | | | | | **nonC4-NADP-ME** | |
| D211 | O^δ1^ | 3.9 | R123 | N^ε^ | D219 | R131 |
| E288 | O^ε1^ | 2.8 | K138(£) | N^ζ^ | E296 | K146 |
| E288 | O^ε2^ | 3.6 | K138(£) | N^ζ^ | E296 | K146 |
| R102(¥) | N^η2^ | 3.7 | E162 | O^ε2^ | R110 | E170 |
| K138(£) | N^ζ^ | 2.8 | E288 | O^ε1^ | K146 | E296 |
| K138(£) | N^ζ^ | 3.5 | E288 | O^ε2^ | K146 | E296 |
| **nonC4-NADP-ME** | | | | | **C4-NADP-ME** | |
| R110(¥) | N^η2^ | 2.7 | E170 | O^ε2^ | R102 | E162 |
| R131 | N^η1^ | 3.5 | D219 | O^δ1^ | R123 | D211 |
| K146(£) | N^ζ^ | 3.8 | E296 | O^ε1^ | K138 | E288 |
| K146(£) | N^ζ^ | 3.7 | E296 | O^ε2^ | K138 | E288 |
| E170 | O^ε2^ | 2.7 | R110(¥) | N^η2^ | E162 | R102 |
| D219 | O^δ1^ | 3.4 | R131 | N^η1^ | D211 | R123 |
| E296 | O^ε1^ | 3.8 | K146(£) | N^ζ^ | E288 | K138 |
| E296 | O^ε2^ | 3.6 | K146(£) | N^ζ^ | E288 | K138 |

**Supplementary Table 6. Stabilizing and destabilizing residues involved in the dimer interface of C4- and nonC4-NADP-ME, identified by PISA analysis.** The analysis includes type of residues involved in dimeric interfaces of protomers A and B of each isoform. Equivalent residues of each isoform are indicated.

| **Stabilizing residues between monomers contacts** | | **Equivalent residue in** |
| --- | --- | --- |
|  | **Residue** | **nonC4-NADP-ME** |
| **C4-NADP-ME** | F289 | F297 |
|  | P202 | P210 |
|  | P128 | P136 |
|  | P127 | P135 |
|  | A129 | A137 |
|  | Y206 | Y214 |
|  |  | **C4-NADP-ME** |
| **nonC4-NADP-ME** | P136 | P128 |
| **Destabilizing residues between monomers contacts** | |  |
|  | **Residue** | **nonC4-NADP-ME** |
| **C4-NADP-ME** | R201 | Q209 |
|  |  | **C4-NADP-ME** |
| **nonC4-NADP-ME** | R171 | T163 |

**Supplementary Table 7. X-ray data collection and refinement statistics for the crystal structure of C4G200R.** *^a^* Statistics for the highest resolution shell are given in parentheses. *^b^* RMS: Root-mean square.

| **Data collection:** |  |
| --- | --- |
| Crystal-detector distance (mm) | 407.3 |
| Rotation range/image (°) | 0.2 |
| No. of frames | 700 |
| Exposure time/image (s) | 0.021 |
| Wavelength (Å) | 0.9762 |
| Space group | *P*2_1_2_1_2_1_ |
| Unit cell parameters |  |
| *a*, *b*, *c* (Å) | 98.68, 124.16, 189.47 |
| *α*, *β*, *γ* (º) | 90, 90, 90 |
| Resolution range (Å) *^a^* | 50.00–2.70 (2.77–2.70) |
| Total reflections | 341,332 (24,753) |
| Unique reflections | 63,889 (4,473) |
| Redundancy | 5.3 (5.5) |
| Completeness (%) | 99.1 (99.8) |
| Mean I/σ(I) | 5.3 (1.1) |
| Overall Wilson *B*-factor (Å^2^) | 50 |
| R_meas_ | 0.213 (1.865) |
| R_pim_ | 0.092 (0.780) |
| CC_(1/2)_ | 0.993 (0.620) |
| Subunits/asymmetric unit | 4 |
| **Refinement:** |  |
| Reflections used in refinement | 60,592 |
| R_free_ test set (%) | 5.0 |
| R_work_ | 0.239 |
| R_free_ | 0.296 |
| No. of non-hydrogen atoms |  |
| all atoms | 17,498 |
| macromolecules | 17,433 |
| ligands | 36 |
| solvent | 29 |
| RMS *^b^* deviations from ideal values |  |
| bonds (Å) | 0.010 |
| angles (º) | 1.71 |
| Average *B*-factor (Å^2^) |  |
| all atoms | 66 |
| protein | 66 |
| ligand | 83 |
| solvent | 35 |
| Ramachandran plot |  |
| favored regions (%) | 94.5 |
| allowed regions (%) | 5.1 |
| outliers (%) | 0.4 |
| **Deposition:** |  |
| PDB code | 9E6M |

**Supplementary Table 8. Hydrogen bond network at the A-C interface of the tetramer in C4-NADP-ME and C4G200R.** PISA analysis of the crystal structures - C4-NADP-ME (PDB 5OU5) and C4G200R (PDB 9E6M) - identified every hydrogen bond linking protomers A and C, specifying the donor/acceptor atoms and the corresponding atom distances (Å). Some contacts are not conserved between the two isoforms, showing the effect of G200R substitution. Isoform-specific contacts are highlighted in green (C4-NADP-ME) and orange (C4G200R).

| **C4-NADP-ME** | | | | | **C4G200R** | | | | |
| --- | --- | --- | --- | --- | --- | --- | --- | --- | --- |
| **Protomer A** |  |  | **Protomer C** |  | **Protomer A** |  |  | **Protomer C** |  |
| L85 | O | 3.8 | R633 | N^η2^ | E72 | O^ε1^ | 3.5 | S617 | N |
| P89 | O | 3.0 | A95 | N | E72 | O^ε1^ | 3.4 | S617 | O^ϒ^ |
| A91 | O | 3.1 | S93 | N | S82 | O | 3.5 | R633 | N^ε^ |
| S93 | O | 2.7 | A91 | N | S82 | O | 3.6 | R633 | N^η1^ |
| N634 | O | 2.9 | V87 | N | P89 | O | 3.1 | A95 | N |
| V87 | N | 2.9 | N634 | O | A91 | O | 3.2 | S93 | N |
| A91 | N | 2.7 | S93 | O | S93 | O | 2.8 | A91 | N |
| S93 | N | 3.0 | A91 | O | N634 | O | 2.9 | V87 | O |
| A95 | N | 2.8 | P89 | O | R633 | N^η2^ | 3.0 | L85 | O |
| R633 | N^η2^ | 3.5 | E83 | O | R633 | N^η2^ | 3.7 | P86 | O |
| N634 | N^δ2^ | 3.4 | E84 | O^ε1^ | R633 | N^η2^ | 3.2 | V87 | O |
| N634 | N^δ2^ | 3.3 | E84 | O | A95 | N | 3.2 | P89 | O |
| R636 | N | 2.7 | V87 | O | S93 | N | 2.9 | A91 | O |
|  |  |  |  |  | S93 | O^ϒ^ | 3.7 | A91 | O |
|  |  |  |  |  | A91 | N | 3.0 | S93 | O |
|  |  |  |  |  | T92 | O^ϒ1^ | 3.8 | P223 | O |
|  |  |  |  |  | V87 | N | 3.2 | N634 | O |

**Supplementary Table 9. Stabilizing and destabilizing residues involved in the tetramer interface of A-C protomers in C4-NADP-ME and C4G200R (PDBs 5OU5 and 9E6M, respectively), identified by PISA analysis.** Isoform-specific contacts are highlighted in green (C4-NADP-ME) and orange (C4G200R).

| **Stabilizing residues between protomers contacts** | |
| --- | --- |
| **C4-NADP-ME** | **C4G200R** |
| P127 |  |
| P89 | P89 |
| A91 | A91 |
| **Destabilizing residues between protomers contacts** | |
| H106 | H106 |
| R633 | R633 |

**Supplementary Table 10. Salt bridge interactions at the A-C interface of the tetramer in C4-NADP-ME and C4G200R.** PISA analysis of the crystal structures - C4-NADP-ME (PDB 5OU5) and C4G200R (PDB 9E6M) - identified every hydrogen bond linking protomers A and C, specifying the donor/acceptor atoms and the corresponding atom distances (Å). C4G200R-specific contacts are highlighted in orange. Two new salt bridge contacts are found due to the G200R substitution. N.R. not salt bridge found.

| **C4-NADP-ME** | | | | | **C4G200R** | | | | |
| --- | --- | --- | --- | --- | --- | --- | --- | --- | --- |
| **Protomer A** |  |  | **Protomer C** |  | **Protomer A** |  |  | **Protomer C** |  |
| N.R. |  |  | N.R. |  | E217 | O^ε1^ | 3.8 | R636 | N^ε^ |
|  |  |  |  |  | E217 | O^ε2^ | 3.4 | R636 | N^η2^ |

**Supplementary Table 11. Hydrogen bond network at the C-D dimer interface of C4-NADP-ME and C4G200R.** PISA analysis of crystal structures 5OU5 (C4-NADP-ME) and 9E6M (C4G200R), in which every protomer adopts the open conformation, identified all hydrogen bonds stabilizing the C-D dimer. For each contact the table lists the interacting residues and atoms together with the heavy-atom distance (Å). Isoform-specific contacts are highlighted in green (C4-NADP-ME) and orange (C4G200R). Residues marked with an asterisk (*) lie in the loop that carries Gly200 in C4-NADP-ME.

| **C4-NADP-ME** | | | | | **C4G200R** | | | | |
| --- | --- | --- | --- | --- | --- | --- | --- | --- | --- |
| **Protomer C** |  |  | **Protomer D** |  | **Protomer C** |  |  | **Protomer D** |  |
| I291 | O | 3.0 | A129 | N | R123 | N^η1^ | 3.2 | K210 | O |
| A129 | N | 3.1 | I291 | O | A129 | N | 3.2 | I291 | O |
| Q146 | O | 3.1 | Q152 | N^ε2^ | N159 | N^δ2^ | 3.2 | N159 | O^δ1^ |
| N159 | O^δ1^ | 3.5 | N159 | N^δ1^ | Q152 | N^ε2^ | 3.3 | Q146 | O |
| N159 | N^δ2^ | 3.4 | N159 | O^δ1^ | Y206 | N | 2.8 | G124 | O |
| G124 | O | 2.8 | Y206 | N | N221 | N^δ2^ | 2.7 | R123 | O |
| R123 | O | 2.9 | N221 | N^δ2^ | K210 | O | 3.3 | R123 | N^η1^ |
| N221 | N^δ1^ | 2.8 | R123 | O | I291 | O | 3.1 | A129 | N |
| Y206 | N | 2.8 | G124 | O | G196 | O | 2.8 | K139 | N^ζ^ |
| Q152 | N^ε2^ | 3.2 | Q146 | O | Q146 | O | 3.2 | Q152 | N^ε2^ |
| R201 | N^η2^ | 3.1 | N159 | O^δ1^ | N159 | O^δ1^ | 3.4 | N159 | N^δ2^ |
| K139 | N^ζ^ | 2.7 | G196(*) | O | G124 | O | 2.7 | Y206 | N |
|  |  |  |  |  | R123 | O | 2.6 | N221 | N^δ2^ |

**Supplementary Table 12. Residues that stabilize or destabilize the C-D dimer interface in C4-NADP-ME and C4G200R.** PISA analysis of the open-state crystal structures, C4-NADP-ME (PDB 5OU5) and C4G200R (PDB 9E6M), classified every interfacial residue as either stabilizing or destabilizing. Isoform-specific residues are colour-coded: green for C4-NADP-ME; orange for C4G200R. Asterisks (*) denote residues located within the Gly200 “G-loop”.

| **Stabilizing residues between protomers contacts** | |
| --- | --- |
| **C4-NADP-ME** | **C4G200R** |
| P127 |  |
| P128 | P128 |
| A129 | A129 |
|  | F199 |
|  | P202(*) |
| Y206 | Y206 |
| F289 | F289 |
| **Destabilizing residues between protomers contacts** | |
|  | R200(*) |
| R201(*) | R201(*) |

**Supplementary Table 13. Salt bridge network at the C-D dimer interface of C4-NADP-ME and C4G200R.** PISA analysis of the open-state crystal structures - C4-NADP-ME (PDB 5OU5) and C4G200R (PDB 9E6M) - identified every inter-subunit ion-pair. For each salt bridge, the table lists the interacting atoms and their separation (Å). Isoform-specific contacts are colour-coded: green for C4-NADP-ME and orange for C4G200R.

| **C4-NADP-ME** | | | | | **C4G200R** | | | | |
| --- | --- | --- | --- | --- | --- | --- | --- | --- | --- |
| **Residue Protomer C** | **Atom** | **Distance (Å)** | **Residue Protomer D** | **Atom** | **Residue Protomer C** | **Atom** | **Distance (Å)** | **Residue Protomer D** | **Atom** |
| E288 | O^ε2^ | 3.3 | K138 | N^ε^ | D211 | O^δ1^ | 2.9 | R123 | N^η1^ |
| E288 | O^ε1^ | 3.2 | K138 | N^ζ1^ | D211 | O^δ1^ | 3.8 | R123 | N^ε^ |
| R102 | N^η2^ | 3.6 | E162 | O^ε2^ | E288 | O^ε1^ | 3.3 | K138 | N^ζ1^ |
| R123 | N^η1^ | 3.8 | D211 | O^δ1^ | K138 | N^ε^ | 2.8 | D211 | O^δ1^ |
| K138 | N^ζ1^ | 3.7 | E288 | O^ε2^ | R123 | N^η2^ | 3.5 | D211 | O^δ1^ |
| K138 | N^ζ1^ | 2.7 | E288 | O^ε1^ | K138 | N^ζ1^ | 2.6 | E288 | O^ε1^ |

**Supplementary Table 14. Hydrogen bond network at the C-D dimer interface of C4G200R- and nonC4- NADP-ME.** PISA analysis of crystal structures C4G200R (9E6M) and AlphaFold 3 nonC4-NADP model, in which every protomer adopts the open conformation, identified all hydrogen bonds stabilizing the C-D dimer. For each contact the table lists the interacting residues and atoms together with the heavy-atom distance (Å). Isoform-specific contacts are highlighted in orange (C4G200R) and yellow (nonC4-NADP-ME). Residues marked with an asterisk (*) lie in the loop that carries Gly200 in C4-NADP-ME.

| **C4G200R** | | | | | **nonC4-NADP-ME** | | | | |
| --- | --- | --- | --- | --- | --- | --- | --- | --- | --- |
| **Protomer C** |  |  | **Protomer D** |  | **Protomer C** |  |  | **Protomer D** |  |
| R123 | N^η1^ | 3.2 | K210 | O | R108 | H | 3.2 | R108 | O |
| A129 | N | 3.2 | I291 | O | A137 | N | 2.9 | I291 | O |
| Q152 | N^ε2^ | 3.3 | Q146 | O | R218 | N^η1^ | 3.8 | P136 | O |
| Y206 | N | 2.8 | G124 | O | Q160 | N^η2^ | 3.7 | 154 | O |
| N221 | N^δ2^ | 2.7 | R123 | O | Y214 | N | 2.6 | G132 | O |
| I291 | O | 3.1 | K129 | N | N229 | N^δ1^ | 2.9 | R131 | O |
| G196 | O | 2.8 | K139 | N^ζ^ | R208 | N^η2^ | 3.7 | Y155 | O |
| Q146 | O | 3.2 | Q152 | N^ε2^ | I291 | O | 2.9 | A137 | N |
| N159 | O^δ1^ | 3.4 | N159 | N^δ2^ | N151 | N^δ2^ | 2.8 | S205 | O |
| G124 | O | 2.7 | Y206 | N | Q154 | O | 3.7 | Q160 | N^ε2^ |
| R123 | O | 2.6 | N221 | N^δ2^ | K147 | N^ζ^ | 2.9 | F207 | O |
| N159 | O^δ1^ | 3.4 | N159 | N^δ2^ | R131 | N^η1^ | 3.9 | R218 | O |
| K210 | O | 3.3 | R123 | N^η1^ | Y214 | N | 2.6 | G132 | O |
|  |  |  |  |  | N229 | N^δ1^ | 2.9 | R131 | O |
|  |  |  |  |  | Y155 | O | 3.7 | R208 | N^η2^ |
|  |  |  |  |  | E170 | O | 3.4 | R110 | N^η2^ |
|  |  |  |  |  | S205 | O | 2.8 | N151 | N^δ2^ |
|  |  |  |  |  | F207 | O | 2.9 | K147 | N^η1^ |
|  |  |  |  |  | R218 | O | 3.9 | R131 | N^η1^ |
|  |  |  |  |  | R110 | N^η2^ | 3.2 | E170 | O |
|  |  |  |  |  | R110 | O | 3.1 | R110 | N^η1^ |
|  |  |  |  |  | P136 | O | 3.8 | R218 | N^η1^ |

**Supplementary Table 15. Residues that stabilize or destabilize the C-D dimer interface of C4G200R- and nonC4- NADP-ME.** PISA analysis of crystal structures C4G200R (9E6M) and AlphaFold 3 nonC4-NADP model structures, classified every interfacial residue as either stabilizing or destabilizing. Isoform-specific residues are colour-coded: yellow for nonC4-NADP-ME and orange for C4G200R. Asterisks (*) denote residues located within the Gly200 “G-loop”.

| **Stabilizing residues between protomers contacts** | |
| --- | --- |
| **nonC4-NADP-ME** | **C4G200R** |
| P136 | P128 |
|  | F199(*) |
|  | Y206 |
|  | P202(*) |
|  | A129 |
|  | F289 |
| **Destabilizing residues between protomers contacts** | |
| R171(*) | R201(*) |
|  | R200(*) |

**Supplementary Table 16. Salt bridge network at the C-D dimer interface of C4-NADP-ME and C4G200R.** PISA analysis of crystal structures C4G200R (9E6M) and AlphaFold 3 nonC4-NADP dimeric model - identified every inter-subunit ion-pair. For each salt bridge, the table lists the interacting atoms and their separation (Å). Isoform-specific contacts are colour-coded: yellow for nonC4-NADP-ME and orange for C4G200R.

| **nonC4-NADP-ME** | | | | | **C4G200R** | | | | |
| --- | --- | --- | --- | --- | --- | --- | --- | --- | --- |
| **Residue Protomer C** | **Atom** | **Distance (Å)** | **Residue Protomer D** | **Atom** | **Residue Protomer C** | **Atom** | **Distance (Å)** | **Residue Protomer D** | **Atom** |
| R131 | N^η1^ | 3.5 | D219 | O^δ1^ | D211 | O^δ1^ | 2.9 | R123 | N^η1^ |
| D219 | O^δ1^ | 3.4 | R131 | N^ε^ | D211 | O^δ1^ | 3.8 | R123 | N^ε^ |
| E296 | O^ε1^ | 3.8 | K146 | N^ζ1^ | E288 | O^ε1^ | 3.3 | K138 | N^ζ1^ |
| E296 | O^ε2^ | 3.6 | K146 | N^ζ1^ | E288 | O^ε2^ | 3.2 | K138 | N^ζ1^ |
| E170 | O^ε2^ | 2.6 | R110 | N^η2^ | R123 | N^η1^ | 2.9 | D211 | O^δ1^ |
| K146 | N^ζ^ | 3.9 | E296 | O^ε1^ | R123 | N^ε^ | 3.8 | D211 | O^δ1^ |
| R110 | N^η2^ | 2.8 | E170 | O^ε2^ |  |  |  |  |  |

**Supplementary Table 17. List of vectors used for recombinant protein expression.** The table includes the name of the encoded protein, the corresponding vector backbone, the method of vector construction (Gene synthesis by BioCat, SDM: site directed mutagenesis, or Gibson cloning), and the *E. coli* strain used as host.

| **Encoded NADP-ME sequence**  **(Name of encoded protein)** | **Vector backbone** | **Production of vector** | **Host**  ***E. coli*** |
| --- | --- | --- | --- |
| **C4-NADP-ME** | pET16b | Alvarez *et al.*, 2019 | Rosetta |
| C4-NADP-ME T92F | pET16b | BioCat | Rosetta |
| C4-NADP-ME F140I | pET16b | Alvarez *et al.*, 2019 | Rosetta |
| C4-NADP-ME N142H | pET16b | BioCat | Rosetta |
| C4-NADP-ME N159D | pET16b | BioCat | Rosetta |
| C4-NADP-ME T163R | pET16b | BioCat | Rosetta |
| C4-NADP-ME D164N | pET16b | BioCat | Rosetta |
| C4-NADP-ME V177E | pET16b | SDM | Rosetta |
| C4-NADP-ME G200R | pET16b | BioCat | Rosetta |
| C4-NADP-ME R201Q | pET16b | BioCat | Rosetta |
| C4-NADP-ME D266R | pET16b | BioCat | Rosetta |
| C4-NADP-ME D304H | pET16b | BioCat | Rosetta |
| C4-NADP-ME F317I | pET16b | BioCat | Rosetta |
| C4-NADP-ME M369V | pET16b | BioCat | Rosetta |
| C4-NADP-ME Q377H | pET16b | BioCat | Rosetta |
| C4-NADP-ME I474V | pET16b | BioCat | Rosetta |
| C4-NADP-ME T506N | pET16b | BioCat | Rosetta |
| C4-NADP-ME A521D | pET16b | BioCat | Rosetta |
| C4-NADP-ME Y632F | pET16b | SDM | Arctic Express |
| C4-NADP-ME +15 | pET16b | BioCat | Rosetta |
| C4-NADP-ME N15 | pET16b | BioCat | Rosetta |
| C4-NADP-ME N15 FI | pET16b | SDM | Rosetta |
| C4-NADP-ME +15 FI | pET16b | SDM | Rosetta |
| C4-NADP-ME NnC4 | pET16b | BioCat | Rosetta |
| C4-NADP-ME +DelN | pET16b | BioCat | Rosetta |
| C4-NADP-ME NnC4 G200R | pET16b | SDM | Rosetta |
| C4-NADP-ME N15_G200R | pET16b | SDM | Rosetta |
| **nonC4-NADP-ME** | pET28b | Alvarez *et al.*, 2019 | Rosetta |
| nonC4-NADP-MEΔ15 | pET16b | BioCat | Rosetta |
| nonC4-NADP-MEΔ15_IF | pET16b | SDM | Rosetta |
| nonC4-NADP-MEΔ15_13aa | pET16b | BioCat | Rosetta |
| nonC4-NADP-MEΔ15_20aa | pET16b | BioCat | Rosetta |
| nonC4-NADP-ME_20aa mut | pET28b | Restriction cloning | Rosetta |
| nonC4-NADP-ME I148F | pET28b | SDM | Rosetta |
| nonC4-NADP-ME R171T | pET28b | SDM | Arctic Express |
| nonC4-NADP-ME N172D | pET28b | SDM | Arctic Express |
| nonC4-NADP-ME R208G | pET28b | SDM | Arctic Express |
| nonC4-NADP-MEΔ15 R171T | pET28b | SDM | Arctic Express |
| nonC4-NADP-MEΔ15 N172D | pET28b | SDM | Arctic Express |
| nonC4-NADP-MEΔ15 R208G | pET28b | SDM | Arctic Express |
| **Cyt2** | pET16b | Gibson cloning | Rosetta |
| Cyt2N | pET16b | Gibson cloning | Rosetta |
| Cyt2+DelN | pET16b | Gibson cloning | Rosetta |

**Supplementary Table 18.** **List of primers used in this study.** The table includes the primer name, sequence, and purpose. Lowercase letters indicate modified nucleotides introduced during site-directed mutagenesis. Bold letters denote the regions of Gibson primers that anneal to the target fragment.

| **Name** | **Sequence 5' to3'** | **Purpose** |
| --- | --- | --- |
| C4 V177E fw | GATAATGTGGaGGAGCTGC | SDM V177E |
| C4 V177E rev | GCAGCTCCtCCACATTATC | SDM V177E |
| C4 Y632F fw | CACTCCCGTCTtCCGCAACTAC | SDM Y632F |
| C4 Y632F rev | GTAGTTGCGGaAGACGGGAGTG | SDM Y632F |
| NC4 R171T fw | CAGGAGAcGAACGAGAGG | SDM R171T |
| NC4 R171T rev | CCTCTCGTTCgTCTCCTG | SDM R171T |
| NC4 N172D fw | CAGGAGAGGgACGAGAGG | SDM N172D |
| NC4 N172D rev | CCTCTCGTcCCTCTCCTG | SDM N172D |
| NC4 R208G fw | CCATCTTTgGtCAACCACAGG | SDM R208G |
| NC4 R208G rev | CCTGTGGTTGaCcAAAGATGG | SDM R208G |
| C4 F140I fw | AATTAAGAAGaTCATGAACACCC | SDM F140I |
| C4 F140I rev | GGGTGTTCATGAtCTTCTTAATT | SDM F140I |
| NC4 I148F fw | AAATTAAGAAGtTCATGCACAAC | SDM I148F |
| NC4 I148F rev | GTTGTGCATGAaCTTCTTAATTT | SDM I148F |
| NC4 A347E fw | TTAATTTGCTTGaAAAATATAGCAA | SDM A347E |
| NC4 A347E rev | TTGCTATATTTTtCAAGCAAATTAA | SDM A347E |
| NC4 E511Q fw | GTACTGCTGAAcAAGCATATAACTG | SDM E511Q |
| NC4 E511Q rev | CAGTTATATGCTTgTTCAGCAGTAC | SDM E511Q |
| NC4 F552L fw | CCTGGATTaGGCCTCGGTC | SDM F552L |
| NC4 F552L rev | GACCGAGGCCtAATCCAGG | SDM F552L |
| C4 G200R fw | CATCTTTcGACGACCACAG | SDM G200R |
| C4 G200R rev | CTGTGGTCGTCgAAAGATG | SDM G200R |
| Cyt2 +N-term fwd 1 | GGAGCAGGAGGAGGCGGAGGTGGCCGCGGCC**GCGGGCGGCGGCGTTGAG** | Add nonC4 N-terminal to Cyt2 step 1 |
| Cyt2 +N-term fwd 2 | ATATCGAAGGTCGTCATATGGCCGCGGAGAT**GGAGCAGGAGGAGGCGGAG** | Add nonC4 N-terminal to Cyt2 step 2 + Cloning Zmcyt2 add Gibson overhangs |
| Cyt2 _fwd gibson | ATATCGAAGGTCGTCATATG**GCGGGCGGCGGCGTTGAG** | Clone Cyt2 add Gibson overhangs |
| Cyt2 _rev gibson | GCTTTGTTAGCAGCCGGATC**TTACCGGTAGCTGCGGTAGATGGGG** | Clone Cyt2 add Gibson overhangs |
| Cyt2 _rev full length | TTACCGGTAGCTGCGGTAGATGG | Clone Cyt2 step 1 add N-terminal |
| Cyt2DelN fw | gcaGGCGtCGtCGTTGAGGAC | Exchange Zmcyt2 N-terminal to nonC4 N-terminal, step 1 |
| Cyt2DelN gibson fw 1 | GGAGCAGGAGGAGGCGGAGGTGGCCGCGGCCGCG**GCAGGCGTCGTCGTTGAGG** | Exchange Zmcyt2 N-terminal to nonC4 N-terminal, step 2 |

**Supplementary Table 19. Cryo-EM data acquisition.**

|  | **nonC4-NADP-ME**  **pH 8.0** | **C4G200R**  **pH 8.0** | **C4G200R**  **pH 4.8** | **C4-NADP-ME**  **pH 8.0** |
| --- | --- | --- | --- | --- |
| Magnification | 215,000 | | | |
| Voltage (kV) | 300 | | | |
| Electron exposure (e^-^/Å^2^) |  | 50 | | |
| Defocus range (μm) |  | -0.8 to -2.6 | | |
| Pixel size (Å) | 0.571 | | | |
| No. of movies | 3,062 | 4,341 | 876 | 1614 |
| No. of initial particle images | 447,513 | 393,438 | 140,065 | 772,117 |
| No. of final particle images | 174,901 | 290,820 | 41,462 | 223,089 |

**Video 1. 3D Overview of maize C4-NADP-ME structure highlighting key loops and residues.** The loops formed by residues T163 and D164 are highlighted in yellow, while the loops containing residues 199-FGRPQG-204 in monomer A and 195-YGSIFGRPQG-204 in monomer B are shown in green. The N- and C-termini are clearly indicated for reference. From the start to 29 seconds, the video displays the overall structure and the relative positions of the highlighted loops, with residues T163, D164, G200, R201, and P202 emphasized in stick representation. Between 29 and 70 seconds, the video illustrates the 3D positioning of these loops in relation to the N- and C-termini, which are colored in different shades of pink and blue, respectively. At the 45-second mark, the spatial relationship between residue Y632 and D164 is specifically highlighted.

**Video 2.** **Overlay of the 3D structures of maize C4-NADP-ME and C4G200R, highlighting key structural differences.** At **14 seconds**, the displacement of the 199-FGRPQG-204 loop is shown for wild-type C4-NADP-ME (colored pink) and C4G200R (colored orange). At **26 seconds**, the video illustrates the relative positioning of differential interactions identified by PISA analysis, including the disrupted Y98-R201 interaction in C4-NADP-ME and the newly formed R123-K210 bond in C4G200R.
